# Bioengineering of air-filled protein nanoparticles by genetic and chemical functionalization

**DOI:** 10.1186/s12951-023-01866-7

**Published:** 2023-03-25

**Authors:** Ram Karan, Dominik Renn, Shuho Nozue, Lingyun Zhao, Satoshi Habuchi, Thorsten Allers, Magnus Rueping

**Affiliations:** 1 King Abdullah University of Science and Technology (KAUST), KAUST Catalysis Center, Thuwal, 23955-6900 Saudi Arabia; 2 Biological and Environmental Science and Engineering, King Abdullah University of Science and Technology (KAUST), Thuwal, 23955-6900 Saudi Arabia; 3 Imaging and Characterization Core Lab, King Abdullah University of Science and Technology (KAUST), Thuwal, 23955-6900 Saudi Arabia; 4 School of Life Sciences, University of Nottingham, Nottingham, NG7 2UH UK; 5 Institute for Experimental Molecular Imaging, University Clinic, RWTH Aachen University, Forckenbeckstrasse 55, 52074 Aachen, Germany

**Keywords:** Halophiles, Extremophiles, Gas vesicle, Nanoparticles, Biomaterials, Bioengineering

## Abstract

**Background:**

Various bacteria and archaea, including halophilic archaeon *Halobacterium* sp. NRC-1 produce gas vesicle nanoparticles (GVNPs), a unique class of stable, air-filled intracellular proteinaceous nanostructures. GVNPs are an attractive tool for biotechnological applications due to their readily production, purification, and unique physical properties. GVNPs are spindle- or cylinder-shaped, typically with a length of 100 nm to 1.5 μm and a width of 30–250 nm. Multiple monomeric subunits of GvpA and GvpC proteins form the GVNP shell, and several additional proteins are required as minor structural or assembly proteins. The haloarchaeal genetic system has been successfully used to produce and bioengineer GVNPs by fusing several foreign proteins with GvpC and has shown various applications, such as biocatalysis, diagnostics, bioimaging, drug delivery, and vaccine development.

**Results:**

We demonstrated that native GvpC can be removed in a low salt buffer during the GVNP purification, leaving the GvpA-based GVNP's shell intact and stable under physiological conditions. Here, we report a genetic engineering and chemical modification approach for functionalizing the major GVNP protein, GvpA. This novel approach is based on combinatorial cysteine mutagenesis within GvpA and genetic expansion of the N-terminal and C-terminal regions. Consequently, we generated GvpA single, double, and triple cysteine variant libraries and investigated the impact of mutations on the structure and physical shape of the GVNPs formed. We used a thiol–maleimide chemistry strategy to introduce the biotechnological relevant activity by maleimide-activated streptavidin–biotin and maleimide-activated SpyTag003-SpyCatcher003 mediated functionalization of GVNPs.

**Conclusion:**

The merger of these genetic and chemical functionalization approaches significantly extends these novel protein nanomaterials' bioengineering and functionalization potential to assemble catalytically active proteins, biomaterials, and vaccines onto one nanoparticle in a modular fashion.

**Graphical Abstract:**

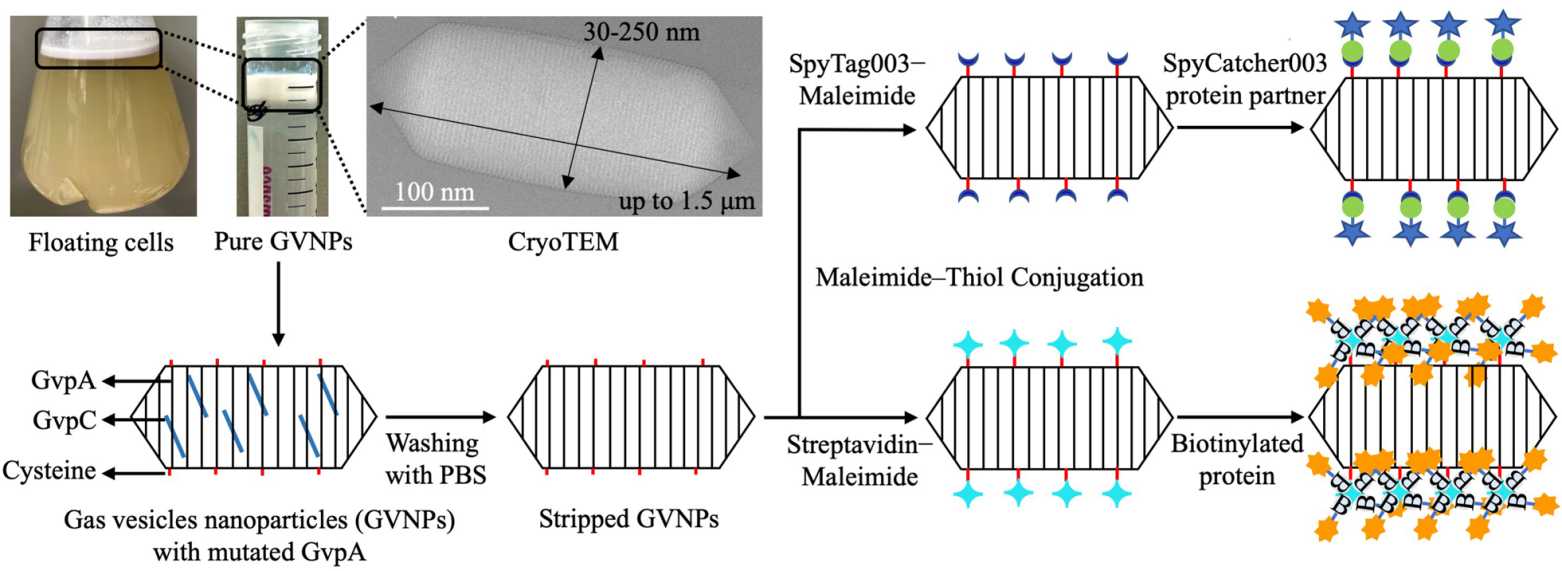

**Supplementary Information:**

The online version contains supplementary material available at 10.1186/s12951-023-01866-7.

## Introduction

Self-assembling protein nanoparticles (NPs) like virus-like particles (VLPs) and gas vesicle nanoparticles (GVNPs) have gained extensive interest as next-generation functional bionanomaterials because of their readily production and unique properties, including modular nature, biocompatibility, and higher-order complexity [1–3]. VLPs are not contagious viral protein complexes with sizes ranging from 20–200 nm [4]. GVNPs are relatively large (widths up to 250 nm and lengths up to 1.5 µm), rigid, low-density hollow intracellular air-filled organelles solely made of protein (lipid or carbohydrate-free), exceptionally stable, inert, nontoxic, self-adjuvants, accessible to bioengineering, and can easily be purified by simple flotation due to their buoyancy [5–7]. The interior surface of the GVNP envelope is hydrophobic and, therefore, water-impermeable, while gases can be exchanged with the surrounding [8–10]. Various aquatic prokaryotes naturally produce GVNPs, enabling them to float in light- and oxygen-rich upper water layers by increasing the microorganism's surface area-to-volume ratio in response to environmental cues [2, 3, 6, 10, 11]. Furthermore, over the past decades, GVNPs have been used in various applications, such as biocatalysis [7], endotoxemia treatment [12], contrast-enhanced ultrasound [13], hyperpolarized xenon magnetic resonance imaging [14], 3D printing [15], and as biocompatible vaccine vectors in vaccine research [9, 12, 16–18].

In-depth studies on haloarchaeal GVNP gene cluster composition and GVNP expression were performed with GVNPs from *Halobacterium* sp. NRC-1 and *Haloferax mediterranei* [7, 9, 17–20]. Depending on the species, 8–14 proteins (Gvp proteins) are required for GVNP biogenesis [6]. In *Halobacterium* sp. NRC-1, 14 gvp genes contribute to the biosynthesis of GVNPs [9, 19]. The GVNPs from *Halobacterium* sp. NRC-1 is valuable for various processes, although one significant challenge is the slow growth rate, expensive antibiotics, and inconsistent induction systems of the *Halobacterium* expression host [6, 15, 21, 22]. Therefore, we recently developed a new, efficient haloarchaeal GVNP expression system using a *Haloferax volcanii* as a host*,* with significantly short growth times that enable the production of haloarchaeal GVNP fusions on a larger scale without the need for antibiotics [15]. Moreover, *H. volcanii* has well-developed microbiological and molecular genetics techniques [23, 24], including a facile DNA transformation system, shuttle plasmids, and selectable markers [25, 26]. The fragile nature of the S-layer cell walls of *H. volcanii* allows for efficient lysis in an aqueous solution, releasing cellular proteins and reducing the cost of protein purification [27–30].

Haloarchaeal GVNP biogenesis genes are organized into two directionally opposing clusters, gvpDEFGHIJKLM upstream and gvpACNO downstream [6, 7, 9]. GvpA and GvpC are the major components of the GVNP complex. The GvpA protein (76 aa) forms the main ribbed structure, and the GvpC protein (380 aa) forms an exterior mesh on the GVNPs surface [6, 9, 11]. The haloarchaeal GVNPs have been bioengineered by fusing several foreign proteins with or within the GvpC protein [7, 9, 12, 16–18, 31]. However, GVNPs from *Halobacterium* sp. NRC-1 tends to lose the GvpC protein during the purification process when performed at lower saline conditions, resulting in the exposure of the ribbed structure formed by GvpA [32]. Pfeifer and co-workers conducted a mutagenesis study on GvpA and showed GvpA viability for the genetic engineering [33, 34].

Inspired by the possibility of bioengineering GvpA, we choose a combinatorial approach to functionalize the GVNPs by the site-specific introduction of cysteines, due to the natural deficiency in GvpA, to facilitate thiol–maleimide reactions. We constructed single, double, and triple variant libraries by selective substitutions of alanine and serine residues to cysteine in exposed loops of GvpA. Further, we extended GvpA by introducing cysteine with linkers at the N- and C-terminus. We examined the impact of insertion and mutations on the structure and physical shape of the GVNPs formed. Additionally, we report the functionalization of the bioengineered GVNPs by the SpyTag003-SpyCatcher003 system [35] and streptavidin to enable the attachment of any recombinant protein conjugated with a biotin tag, supported by thiol–maleimide reactions. These SpyTag003-SpyCatcher003 and streptavidin–biotin GVNP conjugates are diverse, efficient, and powerful tools for GVNPs biotechnological application.

## Materials and methods

### Materials

Gibson Assembly Cloning Kit, restriction enzymes, and Q5^®^ High-Fidelity DNA polymerase were purchased from New England Biolabs (Ipswich, MA, USA). All other chemicals and solvents were purchased from Sigma-Aldrich (St. Louis, MO, USA). *Escherichia coli* (*E. coli*) One Shot^®^ TOP10 chemical competent cells and Measure-IT™ thiol assay kits were purchased from Invitrogen (Karlsbad, CA, USA). The *E. coli* BL21 (DE3) strain has been purchased from Agilent Technologies (Santa Clara, CA, USA), and electrocompetent cells have been prepared according to an in-house protocol. *Halobacterium* sp. NRC-1 was obtained from Carolina Biological Supply (Burlington, NC, USA). We used *Haloferax volcanii* H1895 and its corresponding vector pTA963 in the study [24, 25]. N-terminal maleimides modified SpyTag003 peptide (GGSGGGGRGVPHIVMVDAYKRYK with ≥ 98% purity) was synthesized by GenScript Biotech Corporation, HK.

### GVNP plasmid generation and transformation in *Haloferax volcanii*

*Halobacterium* sp. NRC-1 was cultured in a CM^+^ medium containing 4.3 M NaCl and trace metals at 42 °C with shaking, as previously described [7, 15–17, 36]. The GVNP operon from *Halobacterium* sp. NRC-1 (ATCC 700,922 / JCM 11,081) [37] was PCR amplified from the genome and cloned using the Gibson Assembly Cloning Kit into pTA963 to generate pTA963.GVNP expression plasmid (Additional file 1: Table S1). For the mutagenesis study, the GvpA gene was replaced by a codon-optimized mutated GvpA synthetic gene (GenScript Biotech Corporation, HK or Twist Bioscience, USA). The construct was validated by PCR amplification and DNA sequencing. GVNP-containing vectors were transformed into the *Haloferax volcanii* H1895 using PEG/EDTA method [21].

### Saturation mutagenesis for GvpA

Ab initio protein prediction for gvpA1 (gas vesicle structural protein 1) from *Halobacterium salinarum* (strain ATCC 700,922/JCM 11,081/NRC-1, Uniport: P08958) was performed by I-TASSER [38–41]. Cysteine substitutions were made based on Grantham's distance [42] and Sneath's index [43] to create a combinatorial cysteine mutant library. The initial substituted positions were further analyzed by predicting pairs of residues that will likely form a disulfide bond if mutated to cysteines [44], and dimerization analysis. Based on the GvpA of  *Bacillus megaterium* (PDB: 7R1C), a new model was performed by I-TASSER, creating a more up-to-date model.

### Expression of GVNPs in *Haloferax volcanii*

*H. volcanii* and derivatives were cultured in the Hv-YPC medium containing 4.0 M NaCl at 45 °C with shaking as previously described [25, 28–30, 45]. For solid media, 2% (w/v) agar was added. The processes for producing and culturing GVNPs were performed as previously described [7, 15, 17, 45]. *H. volcanii* lawns or floating cells were grown for 2 weeks and lysed osmotically with PBS solution (137 mM NaCl, 2.7 mM KCl, 10 mM sodium phosphate dibasic, and 2 mM potassium phosphate monobasic, pH 7.4) containing 10 mM MgSO_4_ and 50 μg/mL of DNase I (Sigma-Aldrich, USA). The cell lysate suspension was incubated for 1 h at 37 °C before overnight centrifugation at 60 ×*g* in a swinging bucket rotor in a Sorvall XFR centrifuge (Thermo Scientific, Rockford, IL) to accelerate the floatation of the GVNPs. Intact GVNPs were collected and resuspended in PBS solution, then floated by overnight centrifugation and harvested. This floatation procedure was repeated until a white, milky suspension of GVNPs was obtained. GVNP concentration was quantified via Nanodrop by measuring 2 µl of sonicated and broken GVNPs.

### Dynamic light scattering (DLS)

GVNPs were characterized by dynamic light scattering (DLS) in triplicate using a ZSPNano Zetasizer (Malvern Inc., Malvern, UK) for stability, temperature (20–60 °C), salt (0–4 M NaCl), pH (5.0–12.0) and organic solvents (10%, v/v of glycerol, ethanol, and DMSO). The particle number, diameter, and polydispersity of GVNP populations were determined, and the size distribution values were represented as mean diameter and polydispersity index (PDI). For thermal stability, GVNP suspensions (purified in PBS/2 M NaCl) were incubated in the water bath at different temperatures for up to 48 h. Samples were removed at fixed intervals, cooled at room temperature, and analyzed by dynamic light scattering. To determine the influence of pH and salt concentrations, GVNPs purified in PBS/2 M NaCl were diluted 100 times in different pH/salt concentration buffers. The results were transformed into residual GVNPs, with the control sample on each measurement being set to 100%.

### Western blotting analysis

The methods used were those previously described [7, 17]. Briefly, the SDS-PAGE analysis was performed using the precast Novex^®^ Tris-glycine gels (4–20%, Invitrogen, Carlsbad, CA, USA). Proteins were then transferred to 0.45 μm nitrocellulose membranes (Millipore Corp., Boston, MA). Membranes were blocked for 30 min in Pierce Fast Blocking Buffer (Thermo Fisher Scientific), incubated in blocking buffer supplemented with Anti-GFP Polyclonal Antibody (Thermo Fisher Scientific) or Anti-GvpC antibodies (GenScript, USA), followed by three washing steps with TBST and then incubation with Goat anti-Rabbit IgG (H + L) Cross-Adsorbed Secondary Antibody, Alexa Fluor 488 (Invitrogen, Catalog # A-11008).

### Tryptic digest and LC–MS/MS analysis

The identification of corresponding peptides was performed by LC-MS/MS analysis. The sample (10 μg) was digested with trypsin using the FASP protocol [46]. Peptides were measured using an LTQ-Orbitrap mass spectrometer (Thermo Fisher Scientific, Waltham, MA, USA) and analyzed by MASCOT v2.3 (Matrix Sciences Ltd, UK).

### Fluorescence microscopy imaging

Fluorescence imaging of GVNPs was performed in microfluidic chambers (Ibidi GmbH, sticky-slide VI 0.4) with a cleaned coverslip attached to the bottom. For imaging of GVNP-sfGFP, the coverslip was surface-coated with protein A by incubating with 10 μg ml^−1^ Protein A (Invitrogen) in Hank's Balanced Salt Solution (HBSS) and incubated with 0.1 μg ml^−1^ anti-GvpA antibody (GenScript Biotech Corporation, HK) at 4 °C for 1 h. Finally, the chamber was incubated with 1% (w/v) casein to minimize the nonspecific binding. GVNPs were taken from the concentrated floating portion of the purified GVNP solution and diluted 100 times by PBS or PBS containing 1 M or 2 M NaCl. Then the sample was injected into the chamber. The chamber was flipped with the coverslip side up to promote the interaction with the coated coverslip surface with floating GVNPs. The solution in the chamber was exchanged to remove the unbonded excess after 30 min of incubation.

For the imaging of GVNP-SpyCatcher003-mKate2 (SC.mKate2), the coverslip was incubated with 0.1 mg ml^−1^ poly-L-ornithine hydrobromide (Sigma-Aldrich) for two hours to coat the surface. The GVNPs were taken from the concentrated floating portion of the purified GVNP solution and diluted 100 times. Then the sample was injected into the chamber. The chamber was flipped with the coverslip side up to promote the GVNPs attaching to the coverslip. The solution in the chamber was exchanged to remove the unbonded excess after 5 min of incubation.

Fluorescence imaging experiments were conducted using a home-built wide-field fluorescence microscopy setup with an inverted microscope frame (Olympus, IX71) equipped with a high NA phase-contrast objective lens (Olympus, 100 × oil Ph3 NA = 1.30). A continuous-wave (CW) solid-state laser operating at 488 nm (Cobolt, MLD) was used to excite sfGFP, and 561 nm (Cobolt) was used for mKate2. Imaging was performed by a dichroic mirror (FF506-Di03-25 × 36 for 488 nm excitation and FF580-FDi01 for 561 nm excitation) and mission bandpass filter (FF01-550/88 for 488 nm excitation and FF01-630/92–25 for 561 nm). A fluorescence signal was detected by an EMCCD camera (Andor Technology, iXon3 897) with an exposure time of 300 ms (EM gain of 300). Phase contrast image was measured by the same configuration using a Ph3 condenser annulus plate.

### Electron microscopy measurements

For cryogenic transmission electron microscopy (cryo-TEM), GVNP samples were vitrified by ultra-rapid freezing. Typically, 3 μl of the sample was put on a pre-glow-discharged copper grid coated with a holy carbon layer and blotted from both sides. The blotted grid was plunge-frozen in liquid ethane using Vitrobot Mark IV (ThermoFisher Scientific) and transferred into liquid nitrogen. Samples imaging was performed on Titan Krios G2 (Thermo Fisher Scientific) equipped with a K2 camera (Gatan) placed at the end of the energy filter (Gatan) and operated at 300 kV. Typically, electron doses of ∼50 electrons/ Å^2^ were used.

For room-temperature (RT-)TEM, 4 μl of the purified GVNPs sample was drop casted on pre-glow-discharged carbon film copper grids (Electron Microscopy Sciences) and incubated for 1 min for adsorption. After that, the sample was removed with filter paper. Negative staining was performed by adding 4 µl of 2% uranyl acetate for 1 min. Afterward, the uranyl acetate stain was removed with filter paper, and the grids were air-dried. Images were taken in different magnifications with a US4000 CCD camera (Gatan, Inc., USA) or OneView camera (Gatan, Inc., USA) on a Titan 80–300 (Thermo Fisher Scientific). The lengths and diameters of 25 GVNPs were measured manually in DigitalMicrograph GMS3 (Gatan, Inc., USA) with a selected library.

### Thiol assay

GVNPs variants (20 µM each) were treated with a 50-fold molar excess (1 mM) of a reducing agent, Tris(2-cyanoethyl)phosphine (TCP), for two hours at room temperature with gentle mixing and then dialyzed overnight in 1000 kDa MWCO tubing against PBS. Following the established procedures, the free cysteine GVNPs variant is estimated in triplicate with the Measure-iT™ Thiol Assay kit (M30550, Invitrogen). Triplicate 10 μL GVNP variant samples were assayed; fluorescence was measured at 490/520 nm. The variation (CV) of replicate samples was < 7%.

### Thiol-Maleimide binding

The maleimide-cysteine coupling reaction efficiency of each GVNP-producing variant in 50 mM phosphate buffer, pH 7.0, is tested with thiol-reactive Alexa Fluor 647 C2 maleimide (Thermo Scientific). In this reaction, we mixed GVNPs GvpA variants with the reagent (1:10 mol ratio of estimated cysteine). The reaction proceeded at 4 °C overnight with gentle mixing protected from light. Then the unreacted Alexa Fluor 647 C2 maleimide was removed by overnight dialysis in 1000 kDa MWCO tubing against PBS and three repeated centrifugally assisted flotations. GVNPs retained their milky white appearance during the functionalization process. Labeling efficiencies were assessed by measuring the fluorescence (Ex. 640/ Em. 671 nm) of the GVNP samples.

### Homogeneous fluorescence assay for biotin binding on streptavidin-conjugated GVNPs

The determination of accessible and functional binding pockets of streptavidin (SAV) was done by a fluorescence titration assay using the biotin-4-fluorescein (B4F) dye (Sigma-Aldrich) [47]. The free SAV binding pockets were determined by quenching the fluorescence of the B4F dye upon binding to the SAV pocket. A standard curve was generated using the known amount of commercial SAV protein from *Streptomyces avidinii* (Sigma-Aldrich). The "reverse titration" assay in triplicate was set up in a 96-well plate to estimate the streptavidin binding with GVNPs (20 μM). 20 μl of diluted (60x) streptavidin-conjugated GVNPs were mixed with a fixed amount (8 nM) of B4F (10 μl of 160 nM) and filled up to 200 μl with assay buffer B. After mixing and incubating for 10 min at RT, the fluorescence was measured using Synergy H1 Hybrid Multi-Mode Microplate Reader (BioTek, Winooski, VT) according to standard procedure (490 nm/525 nm, gain 100).

### Thiol-maleimide SpyCatcher003 binding

For thiol-maleimide SpyCatcher003 binding, we mixed GvpA.C variants with the maleimide-modified SpyTag003 peptide (1:10 mol ratio of estimated cysteine) for two hours with gentle mixing at room temperature.

### SpyCatcher003 proteins expression

The SpyCatcher003-mKate2 and SpyCatcher003-Esterase amino acid sequences have been codon optimized for *E. coli* and synthesized by Twist Bioscience, USA, and afterward cloned into pET29b vector (Additional file 1: Figure S1). The respective pET29b_SpyCatcher003-mKate2 and pET29b_SpyCatcher003-Esterase plasmids were transformed into electrocompetent *E. coli* BL21 (DE3). A fresh colony was used to inoculate an overnight LB medium (15 mL) supplemented with kanamycin (50 μg mL^−1^) grown at 37 °C at 180 rpm. 10 mL of the overnight culture was used to inoculate 1 L of LB medium containing kanamycin (50 μg mL^−1^) in a 2 L Erlenmeyer flask at 37 °C at 180 rpm. The recombinant protein expression was induced by adding isopropyl-β-D-1-thiogalactopyranoside (IPTG) (0.5 mM, final) when OD_600_ reached 0.6–0.8. The temperature was lowered to 25 °C, and the culture was incubated for 16 h. The cells were harvested by centrifugation at 4 °C (4000 g, 30 min) and resuspended (1 g in 10 mL) in lysis buffer (100 mM Tris/HCl pH 7.4, 100 mM NaCl, 20 mM imidazole). Before lysis, Pierce™ Protease Inhibitor EDTA-free tablet (Thermo Scientific) was added together with DNase (Sigma Aldrich). Cell disruption occurred using a Cell Disruptor (Constant System Inc.). After 4 °C centrifugation (16,000 g, 20 min), the cleared lysate was used for protein purification by Ni–NTA agarose column (5 mL, GE Healthcare). The protein was eluted by gradient elution with elution buffer (100 mM Tris/HCl pH 7.4, 100 mM NaCl, 500 mM imidazole). The collected fractions were concentrated using an Amicon® Ultra Centrifugal filter (10,000 MWCO). The purified protein was overnight dialyzed against storage buffer (50 mM Tris/HCl pH 7.4, 50 mM NaCl, 5% glycerol) and aliquoted and stored at − 20 °C.

### Biotinylation of horseradish peroxidase

Thermo Scientific Pierce Horseradish Peroxidase (HRP) (Cat. 31,490) was biotinylated with the EZ-Link™ Sulfo-NHS-LC-Biotinylation Kit (Cat. 21,435) according to the supplied procedure. In short, the Sulfo-NHS-LC-Biotin was equilibrated to room temperature, 5 mg of HRP protein was resuspended in 2 mL of PBS, and 30 µl of 10 mM Sulfo-NHS-LC-Biotin was added. The reaction was incubated on ice for two hours. Afterward, Thermo Scientific Zeba Spin Desalting Column was used for separating unreacted NHS-LC-Biotin from biotinylated-HRP.

### Horseradish peroxidase assay

The activity of the horseradish peroxidase (HP) enzyme was determined calorimetrically using 0.3% (w/w) hydrogen peroxide solution (H_2_O_2_), 2.5 mM 4-aminoantipyrine (4-AAP), phenol [48, 49]. Measurements were carried out at room temperature (25 °C) in 200 mM potassium phosphate buffer, pH 7.0, using an ultraviolet (UV)-visible spectrophotometer (Cary 60, Agilent, Santa Clara, CA, USA). We calculated ∆A510/minute from the linear portion of the curve. The decomposition of H_2_O_2_ in μM per minute was referred to in Units. The specific horseradish peroxidase activity is expressed as a unit per milligram of GVNP.

### Esterase activity assay

Esterase activity was measured at 400 nm by spectrophotometer (Cary 60, Agilent, Santa Clara, CA, USA) using 50 mM p-nitrophenyl butyrate in 100 mM sodium phosphate buffer pH 7.2 with 150 mM NaCl at 37 °C [50]. One unit of enzyme activity was defined as the amount of enzyme producing one nanomole of 4-nitrophenol per min. The specific esterase activity is expressed as a unit per milligram of GVNP.

## Result and discussion

### Gas vesicle nanoparticles (GVNPs) expression in *Haloferax volcanii*

**Fig. 1:**
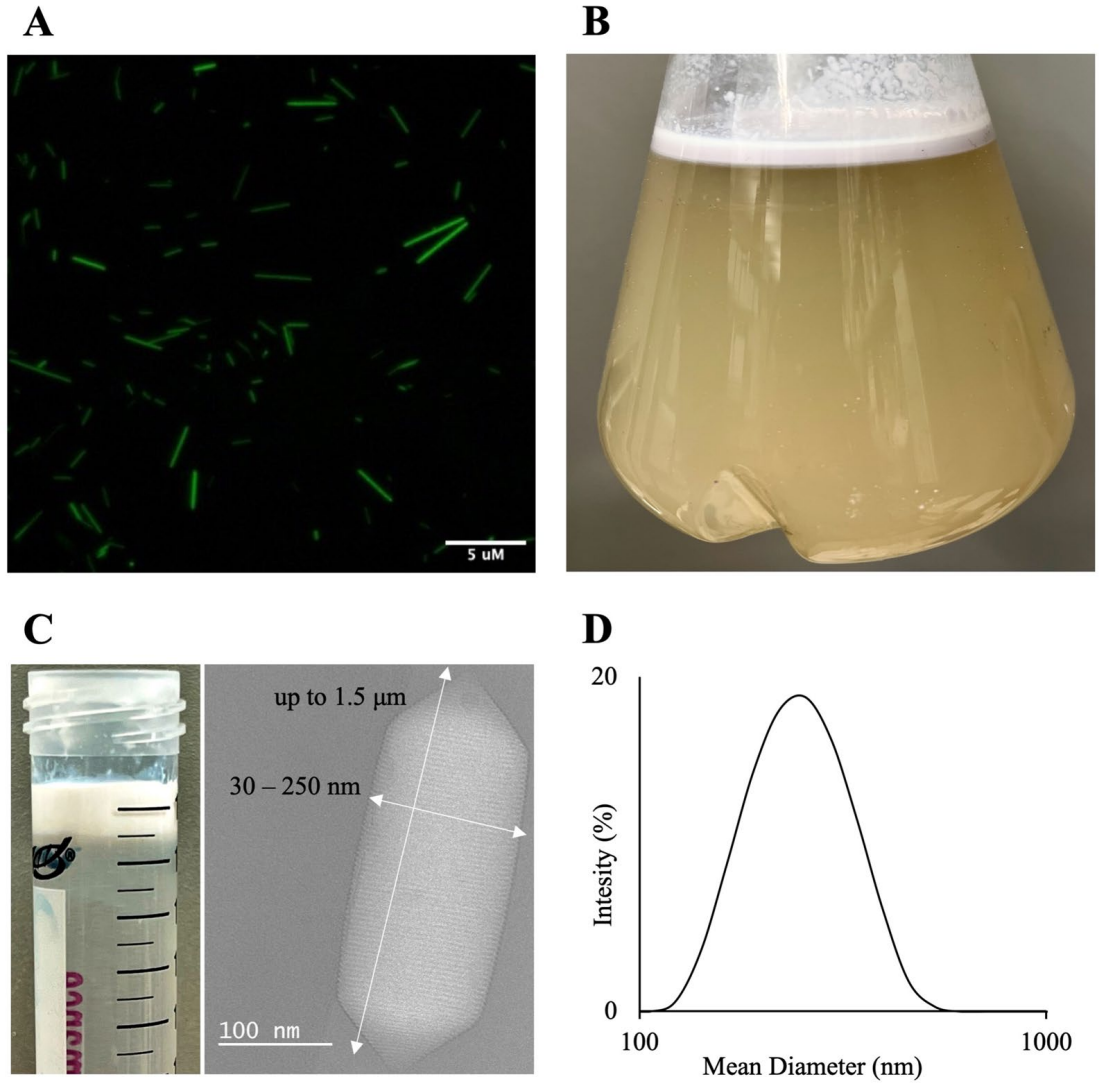
GVNPs engineering and expression in *Haloferax volcanii*. **A** Fluorescing *H. volcanii* cells producing GVNPs fused with sfGFP, **B** Floating *H. volcanii* cells producing GVNPs, **C** Purified recombinant GVNPs and Cryo-TEM image, **D** DLS particle size distribution profile of purified GVNPs

In *Halobacterium* sp. NRC-1, GvpA, and GvpC are the major components of the GVNP membrane. The GvpC protein is located on the outer surface of GVNPs and functions as a "molecular glue" to enhance membrane stability [6, 7, 9]. These characteristics are desirable and potentially crucial for protein display on the surface. Previous studies have suggested that the GvpC protein is sufficiently flexible in tolerating exogenous sequence inserts [7, 16, 20]. We inserted the Haloarchaeal codon-optimized gene of Superfolder Green Fluorescent Protein (sfGFP) into a loop of the acidic tail of the gvpC gene (Additional file 1: Figure S2) and expressed it with the GVNP operon in *Haloferax volcanii* [15]. sfGFP_GVNPs expressing *H. volcanii* cells appear pink and opaque on the plate compared to wild-type red transparent colonies (Additional file 1: Figure S3). The fluorescent signal can be detected in *H. volcanii* cells (Fig. 1A). When cells were grown in liquid culture and left to stand for 1-week, buoyant cells were observed floating at the top (Fig. 1B). Cylinder- or spindle-shaped GVNPs with 30 to 250 nm widths and 40 nm to 1.5 μm lengths with a mean diameter of 255 nm were observed by Cryo-EM (Fig. 1C) and dynamic light scattering (Fig. 1D).

### Establishing a purification scheme for GvpA-only GVNPs

**Fig. 2:**
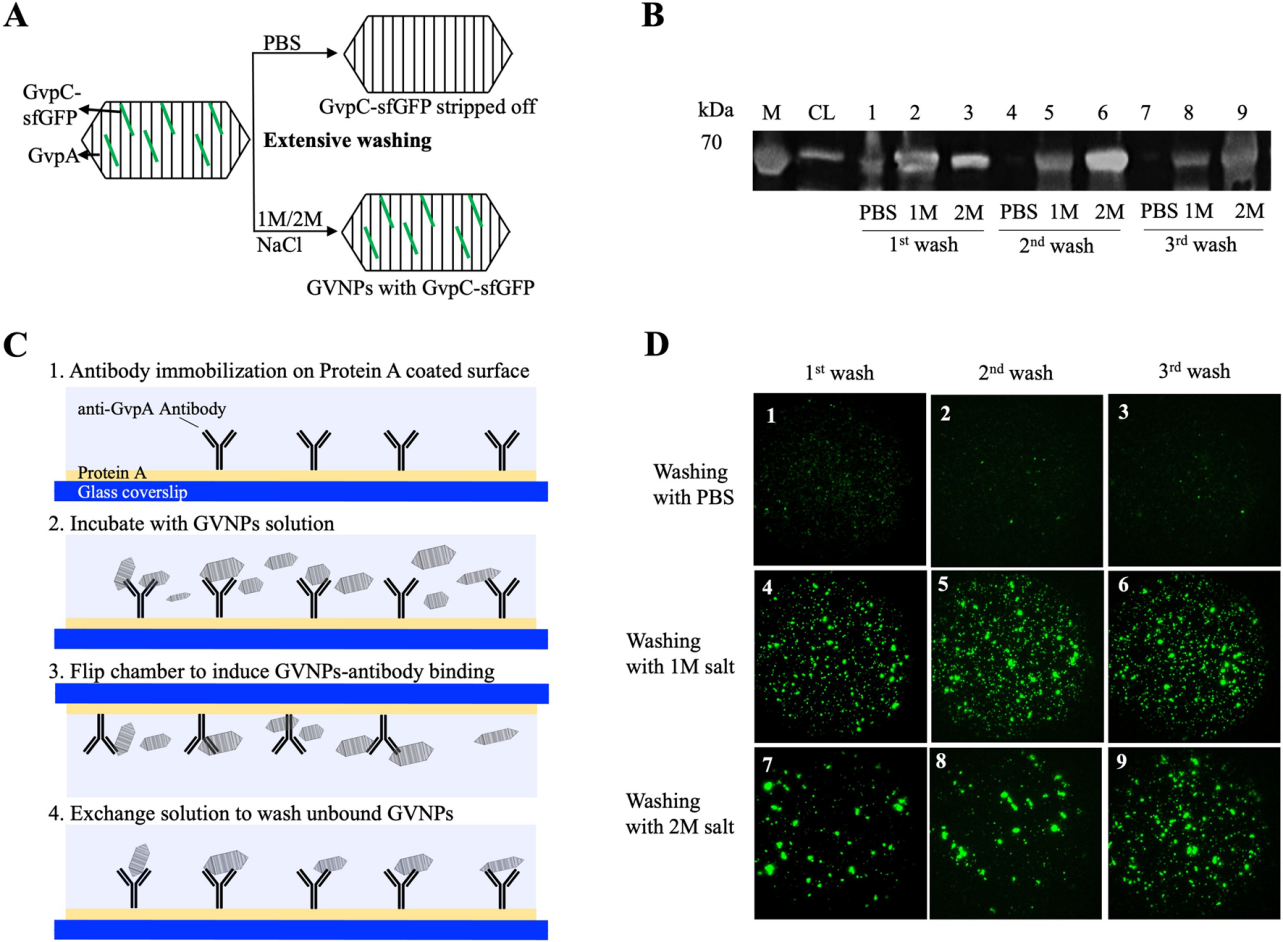
Effect of salt concentrations on GVNP conjugates (GVNP-sfGFP) purification. **A** Schematic illustration describing the procedure, **B** Western blot analysis of GVNPs from each washing condition, **C** Schematic illustration describing the sample preparation procedure to immobilize only GVNPs on coverslip surfaces, **D** sf-GFP fluorescence image of immobilized GVNPs from each washing condition. A small sample was taken from each condition of buffer (PBS, 1 M salt in PBS, 2 M salt in PBS) and the number of washings (1 to 3) and immobilized on the slide chamber. All the fluorescent images were obtained with the same condition (e.g., laser excitation power, detection exposure, etc.). Image contrast is unified for comparison among conditions (image scale 42 $\mu m$ × 42 $\mu m$). Phase-contrast imaging measurement was also performed on the same region of the sample to confirm the presence of GVNPs on the sample

GvpC is an exceptionally acidic (pI 3.57) protein and requires salt for stability. Western blot analysis and fluorescence imaging of GvpC.sfGFP fused GVNPs revealed that GvpC.sfGFP quickly washes off the GVNP surface when GVNPs were purified with a low salt buffer such as PBS. The addition of 1–2 M NaCl to PBS significantly stabilizes the GVNPs during purifications (Fig. 2).

### Characterization of GvpA-only GVNPs

**Fig. 3:**
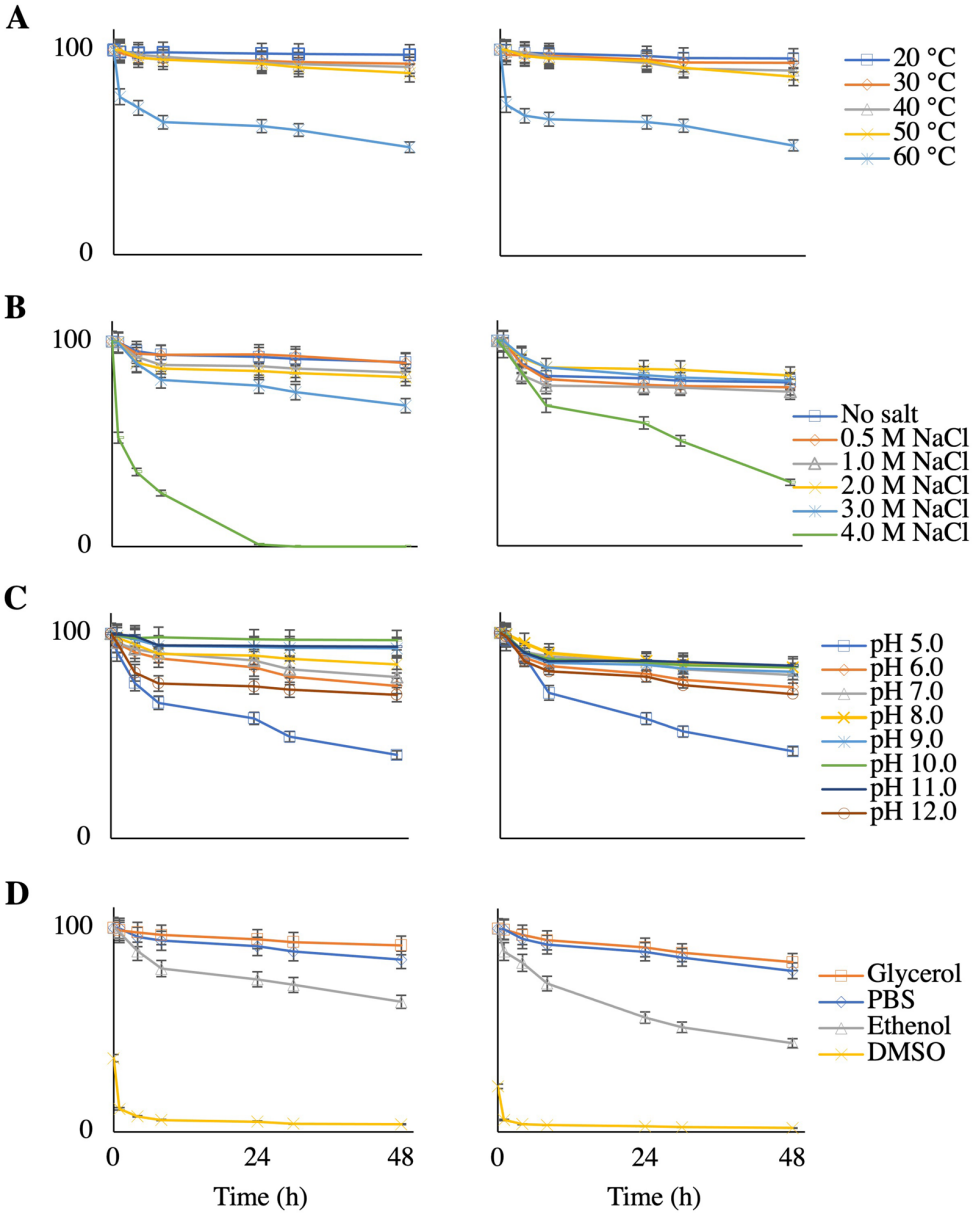
GVNPs characterization (GVNPs with GvpC vs. GvpC stripped-off) **A** Effect of temperature, **B** Effect of salt, **C** Effect of pH, **D** Effect of organic solvents (10%, v/v). Left: GVNPs purified in PBS (GvpC stripped off), Right: 2 M NaCl (containing GvpC). The results were transformed into residual GVNPs (%), with the control sample on each measurement being set to 100%

GvpC protein is reported to stabilize GVNP's exterior surface wall [6]. GvpC was stripped off from the GVNPs, without collapse, by washing with low-salt solutions (Fig. 2, Additional file 1: Figure S4A). Removal of GvpC in the case of cyanobacterial GVNPs reported a marked decrease in the critical collapse pressure of the GVNPs [5, 51, 52], suggesting GvpC provides structural support and removal leads to reducing surface tension pressures. We utilized dynamic light scattering (DLS) to characterize the GVNPs purified in 2 M NaCl (containing GvpC) and PBS (GvpA-only) for salt, thermal, pH, and organic solvent stability (Additional file 1: Figure S4, Fig. 3). GVNPs were serially diluted, straight-line calibrated by DLS, and characterized in the same dilution range (Additional file 1: Figure S4B). Harsh conditions impact the shape and size and break the GVNPs, calculated as kilo counts per second (kcps) (Additional file 1: Figure S4C, D).

GVNPs showed high thermal stability up to 50 °C and degraded fast at 60 °C (Fig. 3A). However, diluted GVNPs purified in PBS are more sensitive at high temperatures, and 80% of GVNPs are degraded within 48 h at 60 °C compared to only 50% degradation in the case of GVNPs purified in 2 M NaCl (Additional file 1: Figure S4E). GVNPs showed exceptional stability in the absence or presence of up to 3 M NaCl. However, 4 M NaCl showed adverse effects, and GVNPs purified in PBS were more sensitive compared to GVNPs purified in 2 M NaCl. Acidic (pH 5.0 and 6.0) and highly alkaline (pH 12.0) solutions harm GNVPs, especially GVNPs purified in PBS (Figs. 3B and C). GVNPs rapidly degrade in DMSO and are sensitive to ethanol. However, glycerol stabilizes GVNPs purified in PBS (Fig. 3D).

To summarize, we demonstrated that native GvpC strips off during the GVNP purification in PBS buffer, leaving the GvpA-based GVNP's shell intact and stable under normal physiological conditions (Fig. 2, Additional file 1: Figure S4A). Interestingly, studies revealed that *A. flos-aquae* GVNPs also become weaker after rinsing off the GvpC; however, GVNPs remained intact over 7 years [11, 51, 53].

Next, we aimed to bioengineer GvpA and selectively introduce cysteines into GVNPs, followed by a thiol-maleimide reaction for functionalization.

### Bioengineering of GvpA 

A single NRC-1 GVNP is predicted to contain around 55,000 GvpA proteins [32]. Moreover, the GvpA protein consists of 76 amino acids (molecular weight 8.0 kDa, theoretical pI: 4.2, Additional file 1: Table S2) [54] and does not require salt for its stabilization according to our measurements (Fig. 2). Previous mutagenesis studies on GvpA demonstrated that GvpA is sensitive to alterations; however, alanine substitutions with non-polar residue at specific regions do not affect the GVNP formation [34]. Subsequently, we performed ab initio protein prediction for GvpA that served as a basis for our cysteine substitutions. The initial library for cysteine introductions in GvpA was further analyzed by predicting possible disulfide bond formations [44]. Considering the antiparallel assembly of the GvpA protein in the GVNP rib structure, next, we analyzed cysteine insertion positions for redundancy due to being located in the possible dimer interface [33, 34]. Following this approach, we created a combinatorial cysteine mutant library of 13 single, 12 double, and 8 triple GvpA variants.

**Fig. 4:**
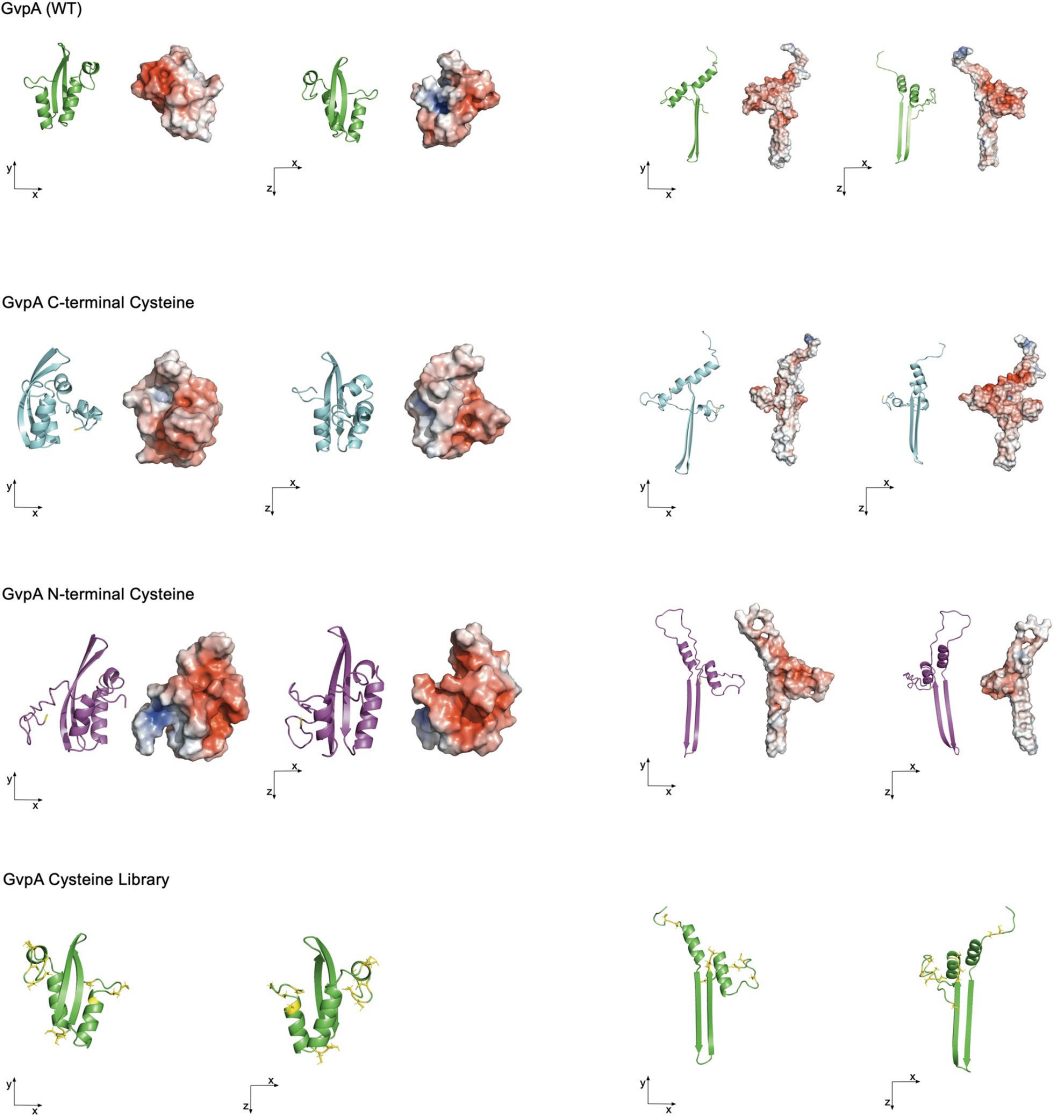
Overview of GvpA cysteine variants. Ab initio protein prediction for gvpA1 (gas vesicle structural protein 1) from *Halobacterium salinarum* (Uniport: P08958) by I-TASSER (left side), and based on GvpA of *Bacillus megaterium* (PDB: 7R1C) (right side). Cysteines are marked in yellow. Surface colors indicate negative and positive electrostatic potentials contoured from 50 kT/e (blue) to –50 kT/e (red). Visualized by PyMOL Molecular Graphics System, Version 2.4.2, Schrödinger, LLC

Furthermore, we investigated the N- or C-terminal viability for genetic engineering of GvpA and introduced cysteine on the N- or C-terminal of GvpA with a short linker (Additional file 1: Table S3). We reevaluated our initial GvpA structural analysis based on the recently published structure of GvpA from *Bacillus megaterium* (PDB: 7R1C) (Fig. 4, see Additional file 1: Figure S5 for sequence alignment) [55]. Our results did not change severely since the structural assumption of two β-sheets and two α-helices was confirmed. Nonetheless, the structural rearrangement of the angles between the two antiparallel β-sheets and the α-helices changed drastically.

**Fig. 5:**
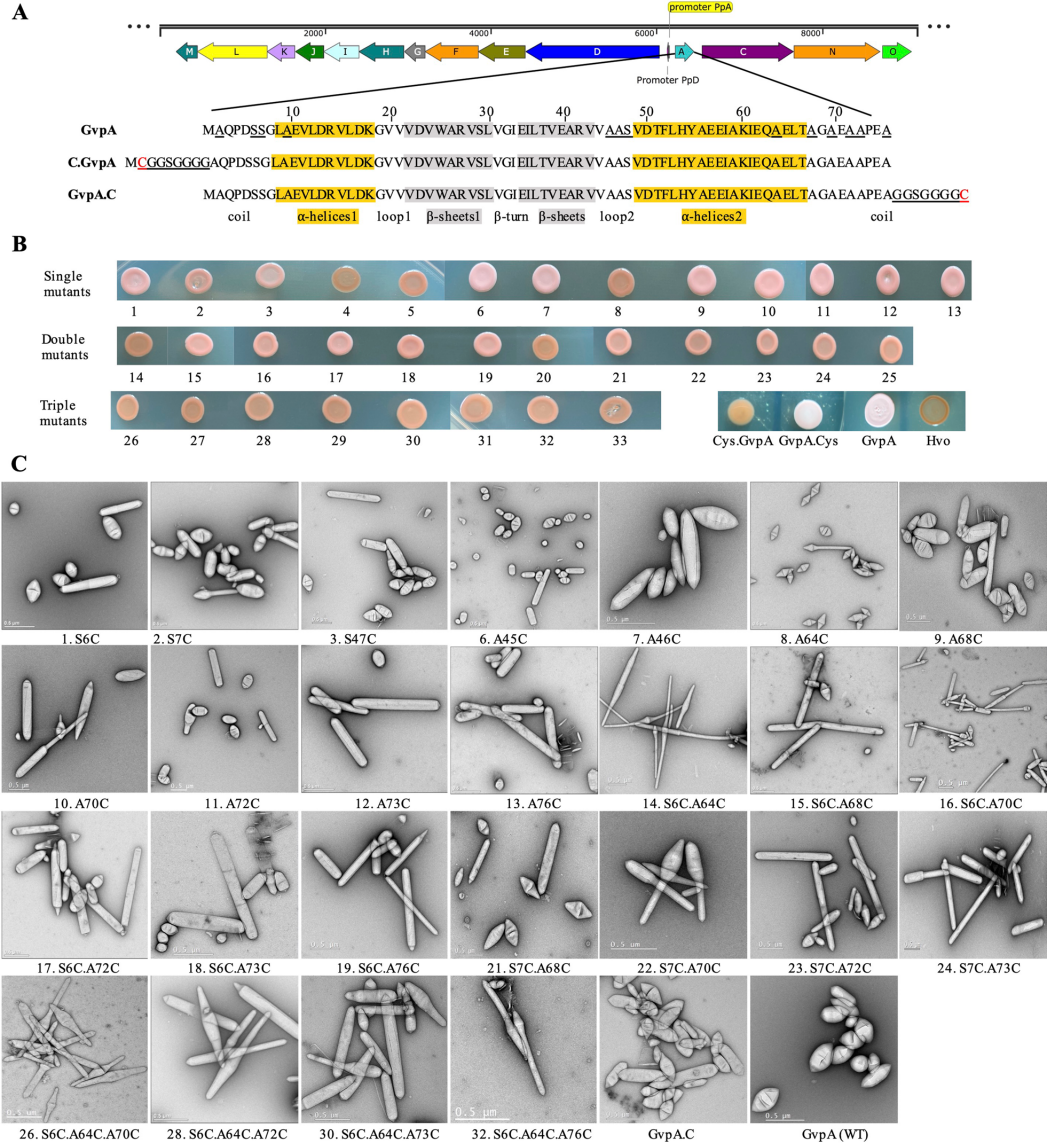
Cysteine mutagenesis within GvpA. **A** The fourteen proteins involved in GVNP formation in *Halobacterium* sp. NRC-1 and amino acid sequences of GvpA (helices α1, α2 highlighted in yellow, β-sheets β1, β2 are in gray) [6, 34], N-terminal and C-terminal cysteine with linker addition in GvpA. GvpA amino acid substitutions are underlined. **B** Phenotype of *H. volcanii* and derivative strains containing GVNP expression plasmids with mutated GvpA. An orange and pink color indicates low and higher levels of GVNPs produced in cells. The numbers indicated variants are detailed in Fig. 5C and Table 1. **C** TEM images of purified GVNPs. The substitutions are shown at the bottom of each panel

The codon-optimized GvpA genes with respective mutations in GvpA were synthesized and replaced the wild-type (WT) GvpA in pTA963.GVNP expression plasmid (Fig. 5A). Each modification was confirmed by PCR and DNA sequence analysis. The resulting plasmids were transformed in *H. volcanii* H1895 and processed for GVNP production as previously described [7, 17, 45]. After 2 weeks, lawns or floating cells were lysed osmotically with PBS solution, and GVNPs were purified by repeated washing in PBS or high salt-containing buffers. The colony phenotype determined the phenotype of *H. volcanii* and derivative strains containing GVNP expression plasmids with mutated GvpA (Fig. 5B). Red or orange colonies on agar plates indicate the abolition of GVNP formation, whereas pink color colonies indicate higher levels of GVNP production in respective transformants. Every transformant was analyzed by tryptic digest, LC–MS/MS, and transmission electron microscopy (TEM) (Fig. 5C, Additional file 1: Figure S6). A summary of these results is presented in Fig. 5 and Table 1. All transformants, except C.GvpA (N-ter), expressed well, evidenced by matched peptides in tryptic digest and LC–MS/MS analysis, even in the case of GVNP negative phenotypes (Additional file 1: Figure S6). However, the addition of cysteine with a linker, GGSGGGG, on the N-terminal of GvpA abolishes GVNP formation. In tryptic digest analysis, we found most GVNPs forming proteins, including GvpC, but we could not recognize any peptide of C.GvpA (N-ter). Surprisingly, similar modifications on the C-terminal of GvpA (GvpA.C) form GVNPs similar to wild-type (Fig. 5A). Several studies revealed that GvpA forms the essential core of the GVNP structure and is necessarily required to produce GVNPs [3, 19, 34]. Therefore, (i) non-production of a GvpA e.g., C.GvpA (N-ter) variant, or (ii) mutations close to the N-terminal of GvpA e.g., A2C, abolishes the GVNP formation. Among 33 GvpA variants (13 single,12 double, and 8 triple variants), eight transformants (two single variants, A2C and A10C, two double variants, S7C.A64C, S7C.A76C, and five triple variants, S7C.A64C.A70C, S7C.A64C.A72C, S7C.A64C.A73C, S6C.A64C.A76C, S7C.A64C.A76C) resulted to GVNP negative cells (Vac-) —colonies were translucent and red —and the other 25 resulted to gas vesiculation (Vac +)—colonies appeared turbid and pink-white (Fig. 5B). The diameters and lengths of these purified GVNPs were also determined by TEM for 25 particles each (Table 1). Pfeifer and co-workers performed a mutagenesis study on GvpA and demonstrated that GvpA tolerates alanine-to-serine substitutions at position 10 (A10S), resulting in GVNPs similar to wild-type [34]. However, here A10C resulted in the lack of GVNP, suggesting that cysteine is involved in disulfide bond formation and negatively impacts the GVNP formation.

**Table 1:** Cysteine mutagenesis within GvpA and effect on GVNPs formation

Variant	GVNPs phenotype	Diameter (µm)		Average (µm)	Width (µm)		Average (µm)
		min	max		min	max	
GVNP (WT)	Spindle/cylinder	0.26	0.83	0.438	0.11	0.27	0.207
1.S6C	Spindle/cylinder	0.15	1.04	0.450	0.13	0.26	0.191
2.S7C	Spindle/cylinder	0.24	1.35	0.557	0.13	0.27	0.179
3.S47C	Spindle/cylinder	0.31	1.4	0.632	0.13	0.31	0.228
4.A2C	Negative	–	–	–	–	–	–
5.A10C	Negative	–	–	–	–	–	–
6.A45C	Spindle/cylinder	0.24	1.61	0.532	0.17	0.29	0.224
7.A46C	Spindle/cylinder	0.17	1.02	0.503	0.11	0.25	0.171
8.A64C	Mini spindle	0.21	1.09	0.354	0.10	0.15	0.119
9.A68C	Spindle/cylinder	0.38	2.03	0.733	0.15	0.28	0.227
10.A70C	Spindle/cylinder	0.35	2.06	0.746	0.12	0.27	0.209
11.A72C	Spindle/cylinder	0.34	1.31	0.730	0.14	0.33	0.243
12.A73C	Cylinder	0.17	1.72	0.755	0.17	0.23	0.182
13.A76C	Cylinder	0.23	2.04	1.095	0.14	0.18	0.162
14. S6C.A64C	Spindle/longer	0.35	2.76	1.851	0.13	0.21	0.156
15. S6C.A68C	Cylinder/longer	0.18	2.72	1.259	0.09	0.25	0.155
16. S6C.A70C	Spindle/cylinder/longer	0.45	1.65	1.024	0.10	0.22	0.146
17. S6C.A72C	Spindle/cylinder/longer	0.37	1.86	0.972	0.14	0.25	0.188
18. S6C.A73C	Cylinder/longer	0.19	2.36	0.974	0.10	0.21	0.142
19. S6C.A76C	Cylinder/longer	0.49	1.73	1.049	0.13	0.21	0.166
20. S7C.A64C	Negative	–	–	–	–	–	–
21. S7C.A68C	Spindle/cylinder/longer	0.26	1.91	0.682	0.09	0.25	0.175
22. S7C.A70C	Spindle/cylinder	0.36	1.54	0.890	0.08	0.19	0.138
23. S7C.A72C	Spindle/cylinder/longer	0.14	2.04	0.711	0.08	0.19	0.151
24. S7C.A73C	Cylinder/longer	0.32	1.88	1.028	0.09	0.24	0.158
25. S7C.A76C	Negative	–	–	-	–	–	–
26. S6C.A64C.A70C	Spindle/longer	0.22	2.36	1.015	0.05	0.19	0.125
27. S7C.A64C.A70C	Negative	–	–	–	–	–	–
28. S6C.A64C.A72C	Spindle/longer	0.29	2.73	1.226	0.10	0.21	0.171
29. S7C.A64C.A72C	Negative	–	–	–	–	–	–
30. S6C.A64C.A73C	Spindle/cylinder/longer	0.24	1.38	0.759	0.11	0.23	0.162
31. S7C.A64C.A73C	Negative	–	–	–	–	–	–
32. S6C.A64C.A76C	Spindle/longer	0.23	2.33	1.119	0.06	0.17	0.104
33. S7C.A64C.A76C	Negative	–	–	-	–	–	–
C.GvpA (N-ter)	Negative	–	–	-	–	–	–
GvpA.C (C-ter)	Spindle/cylinder	0.18	0.80	0.444	0.10	0.23	0.157

Spindle-shaped: a circular cross-section and tapering towards each end; spindle/cylinder: mixed populations

Single mutations in GvpA, S6C, S47C, A45C, A46C, A68C, A70C, and A72C resulted in the spindle and cylinder-shaped GVNPs, like wild types; however, they were comparatively bigger (except S6C). Interestingly, the A64C transformant's GVNPs were tiny and mini-spindle-shaped, suggesting that the alteration disturbed the enlargement of the GVNP structure. On the other hand, A73C and A76C were bigger and cylinder-shaped. Most GvpA variants' GVNP populations' showed a broad distribution in shape and size, especially A68C, A70C, A73C, and A76C. Strunk et al. (2011) reported that deletion of 5 or 7 amino acids at the C-terminus of GvpA did not affect the GVNP formation and resulted in wild-type GVNPs, suggesting that the C-terminus of GvpA can tolerate alteration [56].

GVNPs from double and triple variants were predominantly longer and contained mixtures of spindle and cylindrical-shaped. Interestingly, double or triple variants of S6C and A64C transformant's GVNPs were spindle and longer, with an average diameter of 1.85 μm. A few GVNPs were more than 2 μm in length. However, all the double or triple variants of S7C and A64C resulted in GVNP-negative cells (Vac-).

Only GVNPs that were formed successfully and stable were used as a basis for the functionalization studies.

### Functionalization of cysteine-GvpA only GVNPs

We introduced cysteine into a specific site along the GvpA protein amino acid chain to functionalize the GVNPs by bioconjugation with maleimides. The native GvpC layer was almost entirely removed by extensive washing of GVNPs in PBS before GvpA-cysteine functionalization (Fig. 2). Next, we estimated free cysteine on the GVNP variant and examined the GvpA variant's GVNP maleimide-cysteine coupling efficiency.

### Detection of free thiols in intact cysteine-GvpA only GVNPs

**Fig. 6:**
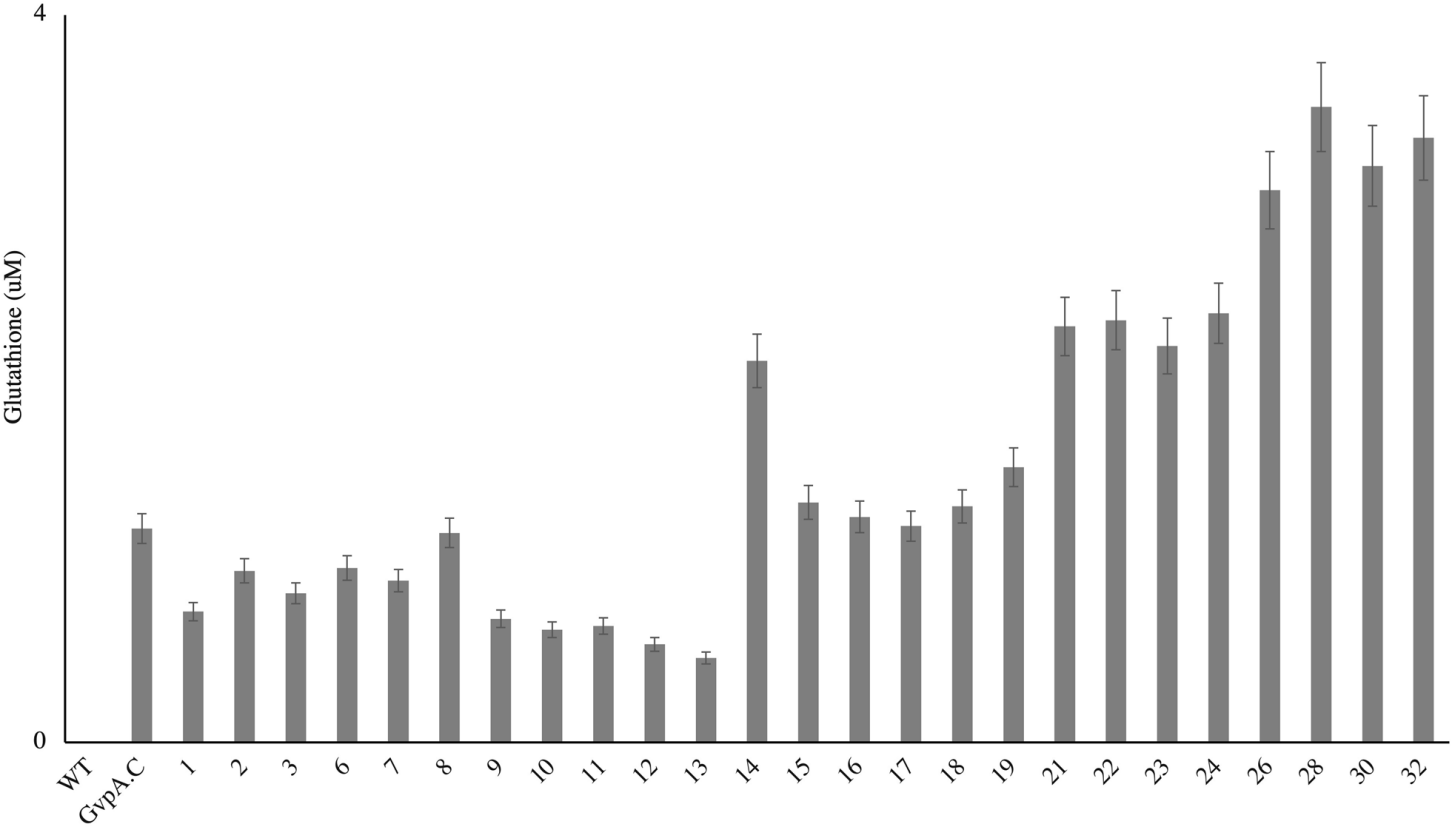
Detection of free thiols in intact GVNPs. Triplicate GVNP variant samples were assayed. The variation (CV) of replicate samples was < 7%. The number corresponds to different constructs described in Table 1 and Fig. 5

Detection of free thiols in intact GVNPs can indicate cysteine residues accessible for maleimide conjugation. The free cysteines in the GVNPs variant are estimated in triplicate measurements with the Measure-iT™ Thiol Assay kit (Invitrogen) by following the producer's recommendations (Fig. 6). The amount of cysteine among the single variants varies from 0.46 µM (A76C) to 1.18 µM (GvpA.C). Interestingly, compared to single variants, cysteine was estimated about 2–3 times in double (1.3–2.3 µM) and triple variants (3.0–3.5 µM).

### Binding reactivity with a small fluorophore

**Fig. 7:**
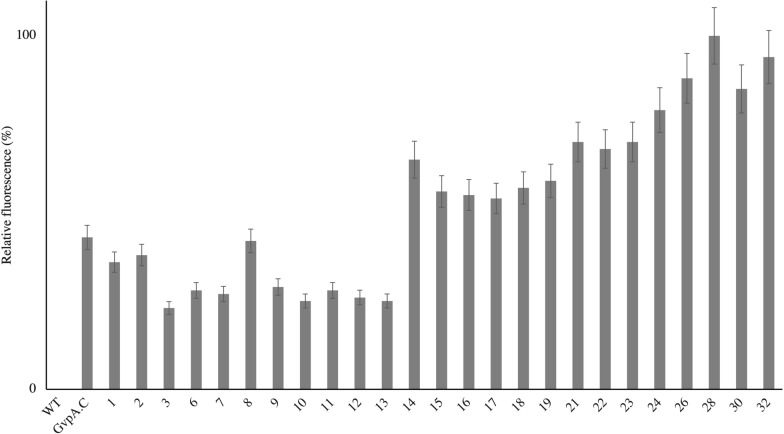
GvpA variants GVNPs binding reaction with Alexa Fluor 647 C2 Maleimide. Fluorescence was measured at 640/671 nm. The variation (CV) of replicate samples was < 8%. The number corresponds to different constructs described in Table 1 and Fig. 5

The maleimide-cysteine coupling reaction efficiency of each GVNP is tested with thiol-reactive Alexa Fluor 647 C2 maleimide. In this reaction, we mixed GVNPs GvpA variants with the reagent (1:10 mol ratio of estimated cysteine). GVNPs retain their milky white appearance during the functionalization process. Labeling efficiencies were assessed by measuring the fluorescence intensity of the GVNP samples (Fig. 7). Wild-type GVNPs treated similarly were used as a control. The highest fluorescence was recorded for triple mutated GvpA variants, followed by double and single GvpA variants.

Interestingly, double and triple-mutated GvpA variants show almost double and triple fluorescence compared to the single variants. Among the single variants, A64C and GvpA.C (C-ter) shows the highest coupling reaction. Because of the flexible linker cysteine of GvpA.C (C-ter) GvpA variant is possibly more accessible for the thiol reaction.

GvpA.C variant produces GVNPs like the wild type; therefore, used for further coupling applications. After demonstrating the ability of GvpA variants to serve as a cysteine-mediated GVNP functionalization platform, we examined the GvpA.C variant's GVNPs' capacity for maleimide chemical conjugation. As the first proof of concept, we tested maleimide-activated streptavidin, biotinylated horseradish peroxidase conjugation, and maleimide-activated SpyTag003 and SpyCatcher003 system conjugation.

### Maleimide-activated streptavidin and biotinylated horseradish peroxidase

**Fig. 8:**
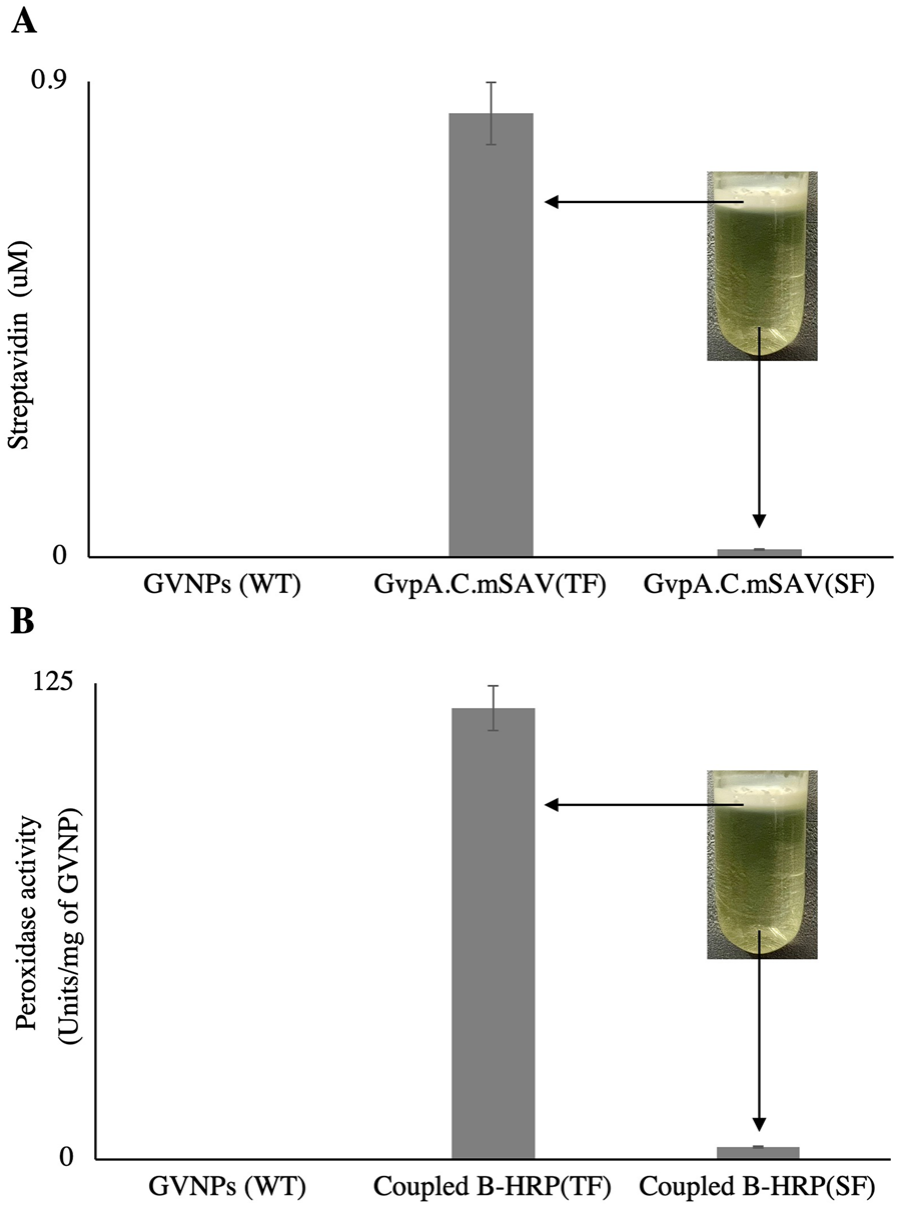
Maleimide-activated streptavidin and biotinylated horseradish peroxidase **A** Streptavidin estimation in GVNPs by titrating it with B4F, **B** Horseradish peroxidase enzyme activity in GVNPs. TF: Top fraction, SF: subnatant fraction

The biotin-streptavidin binding is known for specificity and high affinity and is frequently used [57]. Here, we conjugated maleimide-activated streptavidin with the GvpA.C variant GVNPs (20:1 mol ratio of free cysteine in the GVNPs) at 4 °C overnight with gentle mixing. The free maleimide-activated streptavidin molecules were removed by repeated GVNPs floatation, and bound streptavidin quantity was measured by Biotin-4-Fluorescein (B4F) based fluorescence quenching assay (Additional file 1: Figure S7). In the reduced state, each free thiol group of GVNPs appears to react with a single molecule of maleimide-activated streptavidin (0.8 μM streptavidin in 20 μM of GVNPs, Fig. 8A).

Next, the streptavidin-conjugated GVNPs were incubated overnight at 4 °C in PBS buffer with biotinylated horseradish peroxidase (B-HRP) at an 80-molar excess. The B-HRP-streptavidin-GVNP complex was purified by three repeated centrifugally assisted floatation and overnight dialysis in 1000 kDa MWCO tubing against PBS before horseradish peroxidase assay. Streptavidin on GVNPs coupled well with B-HRP and detected by high horseradish peroxidase activity in the floating top fractions (TP) containing the GVNPs-B-HRP complex compared to centrifugation-separated subnatant fraction (SF) (Fig. 8B).

These results showed that we could successfully functionalize the streptavidin-conjugated GVNPs with biotinylated horseradish peroxidase.

### Maleimide-activated SpyTag003- SpyCatcher003 functionalization of GVNPs

**Fig. 9:**
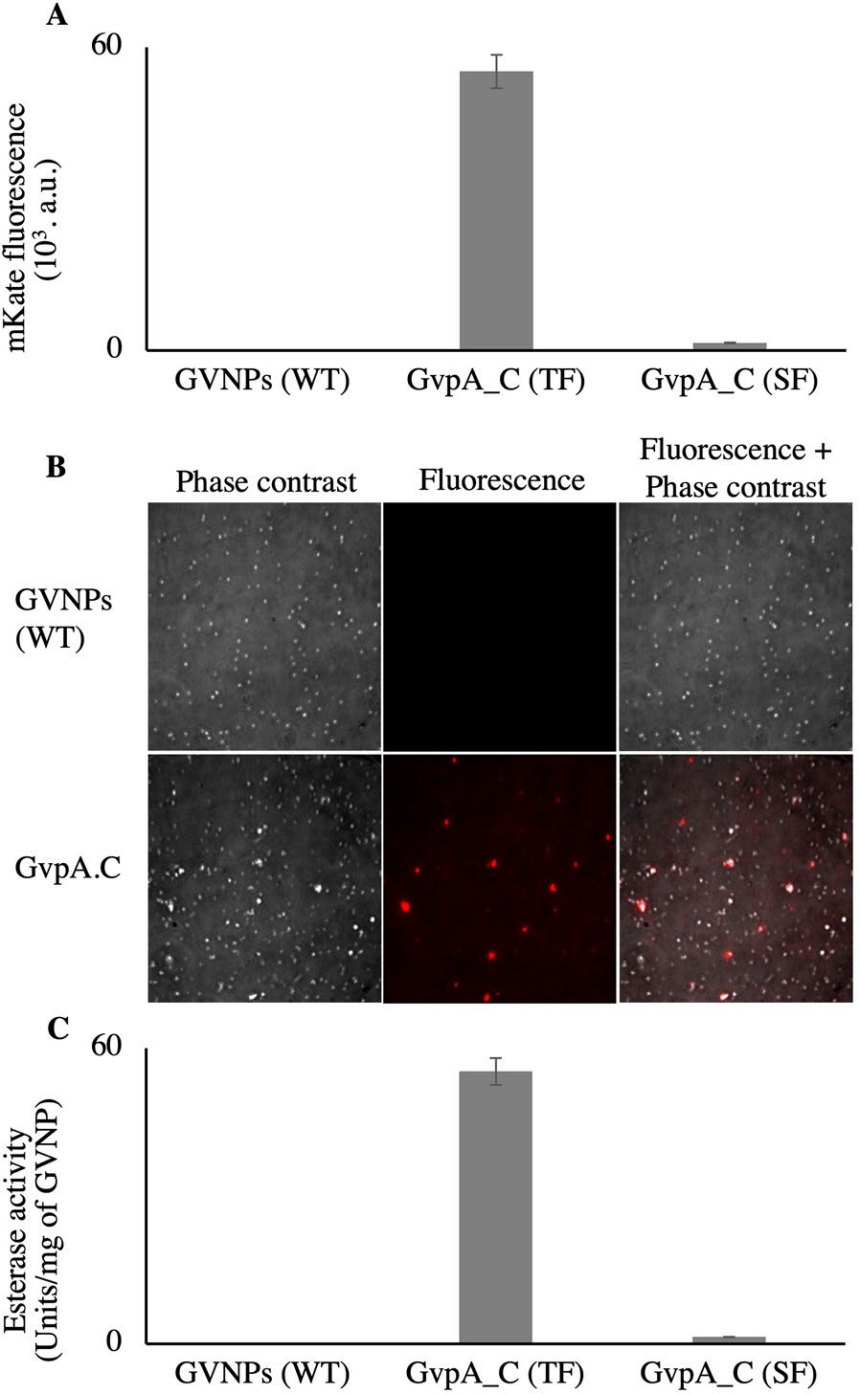
Maleimide-activated SpyTag003-SpyCatcher003 functionalization of GVNPs. **A** mKate2 fluorescence (588/633 nm) of maleimide-activated SpyTag003- mKate2.SpyCatcher003, **B** Phase contrast and fluorescence images of the maleimide-activated SpyTag003- mKate2.SpyCatcher003 (bottom) and the GVNPs (top) (Image scale 38 $\mu m$ × 38 $\mu m$), **C** Estimation of GVNPs bound esterase activity. TF: Top fraction, SF: subnatant fraction.

To simplify GVNP functionalization, the SpyTag003/SpyCatcher003 protein conjugation under physiological conditions system was employed. Noteworthy, the system is versatile, works over a broad range of reaction conditions, and is flexible in the fusion sites [35]. As a proof of concept, the GVNP-maleimide-activated SpyTag003 complex is conjugated with a recombinantly expressed red fluorescent protein, SpyCatcher003-mKate2 (SC.mKate2). The resulting floating fraction GVNPs-SC.mKate2 complex gives high fluorescence compared to centrifugation-separated subnatant fraction (SF) (Fig. 9A). mKate2 colocalizes with GVNPs seen as fluorescent dots and perfectly superimposed with GVNPs appear on the phase contrast image (Fig. 9B).

The resulting GVNP conjugated complexes were purified by repeated buoyancy enrichment and overnight dialysis in 1000 kDa MWCO tubing against PBS before fluorescence measurement or esterase assay (Fig. 9C). The esterase activity shows a successful binding. This improved system allows for faster functionalization of GVNPs in a more biocompatible way.

## Conclusion

Here we describe the development of a new genetic and chemical approach for efficient and selective modification of the GvpA-only GVNP surface by cysteine residues. Serine or alanine residues in GvpA are selected based on the GvpA model analysis and replaced with cysteine to create a variant library of 13 single, 12 double, eight triple, and two N- or C-terminal modified GvpA variants. We examined the resulting GvpA variants for their impact on the GVNP formation, structure, and physical shape. The site-directed mutated GvpA proteins were efficiently and selectively labeled via cysteine-maleimide mediated reaction at physiological conditions and tested for foreign protein display on the GVNP surface via streptavidin-biotin or SpyTag003-SpyCatcher003 conjugation. In summary, the merger of these genetic and chemical approaches enabled a marked increase in GVNP's functional diversity and applicability for various medical, environmental, and biotechnological applications.

## Supplementary Information

**Additional file 1: Table S1.** Oligonucleotides used in this study. **Table S2.** Amino acid composition of GvpA. **Table S3. **Cysteine site-saturation mutagenesis within GvpA. **Figure S1:** Amino acid composition and plasmids of SpyCatcher003-mKate2 and SpyCatcher003-esterase. **Figure S2.** Arrangement of gvp genes in *Halobacterium* sp. NRC-1. **Figure S3.** Gas vesicle nanoparticles (GVNPs) engineering and expression in *Haloferax volcanii*. **Figure S4.** Gas vesicles characterization (GVNPs with GvpC vs. GvpC stripped-off). **Figure S5.** Sequence alignment gvpA1 (gas vesicle structural protein 1) from *Halobacterium salinarum* (Uniport: P08958) and GvpA of *Bacillus megaterium* (PDB: 7R1C). **Figure S6.** Tryptic digest and LC-MS/MS analysis of GvpA mutants and GVNP conjugates. **Figure S7.** Quenching of biotin-4-fluorescein (B4F) fluorescence by adding various amounts of pure streptavidin. **Discussion 1** C.GvpA (N-ter). **Discussion 2** GVNP morphology changes and cysteine influence. **Figure S8.** Pseudo-atomic model of an entire GVNP particle. **Figure S9** GVNP subunit with GvpA models based on the pseudo-atomic model of an entire GVNP particle by Huber et al.

## Data Availability

The datasets generated during the current study are available from the corresponding author upon reasonable request.
