# Supplementary material for: Bioengineering of air-filled protein nanoparticles by genetic and chemical functionalization
Source: J Nanobiotechnology. 2023 Mar 25;21:108. doi: 10.1186/s12951-023-01866-7 (PMC10039352; doi:10.1186/s12951-023-01866-7)
Supplement: Supplementary file 1 — Additional file 1: Table S1. Oligonucleotides used in this study. Table S2. Amino acid composition of GvpA. Table S3. Cysteine site-saturation mutagenesis within GvpA. Figure S1: Amino acid composition and plasmids of SpyCatcher003-mKate2 and SpyCatcher003-esterase. Figure S2. Arrangement of gvp genes in Halobacterium sp. NRC-1. Figure S3. Gas vesicle nanoparticles (GVNPs) engineering and expression in Haloferax volcanii. Figure S4. Gas vesicles characterization (GVNPs with GvpC vs. GvpC stripped-off). Figure S5. Sequence alignment gvpA1 (gas vesicle structural protein 1) from Halobacterium salinarum (Uniport: P08958) and GvpA of Bacillus megaterium (PDB: 7R1C). Figure S6. Tryptic digest and LC-MS/MS analysis of GvpA mutants and GVNP conjugates. Figure S7. Quenching of biotin-4-fluorescein (B4F) fluorescence by adding various amounts of pure streptavidin. Discussion 1 C.GvpA (N-ter). Discussion 2 GVNP morphology changes and cysteine influence. Figure S8. Pseudo-atomic model of an entire GVNP particle. Figure S9 GVNP subunit with GvpA models based on the pseudo-atomic model of an entire GVNP particle by Huber et al. [file 12951_2023_1866_MOESM1_ESM.docx]

**­­­ SUPPLEMENTARY INFORMATION**

**Bioengineering of air-filled protein nanoparticles by genetic and chemical functionalization**

Ram Karan^1^*, Dominik Renn^1^, Shuho Nozue^2^, Lingyun Zhao^3^, Satoshi Habuchi^2^, Thorsten Allers^4^, Magnus Rueping^1,5^*

^1^King Abdullah University of Science and Technology (KAUST), KAUST Catalysis Center, Thuwal 23955-6900, Saudi Arabia

^2^King Abdullah University of Science and Technology (KAUST), Biological and Environmental Science and Engineering, Thuwal, 23955-6900, Saudi Arabia

^3^King Abdullah University of Science and Technology (KAUST), Imaging and Characterization Core Lab, Thuwal, 23955-6900, Saudi Arabia

^4^ University of Nottingham, School of Life Sciences, Nottingham NG7 2UH, UK

^5^Institute for Experimental Molecular Imaging, University Clinic, RWTH Aachen University, Forckenbeckstrasse 55, D52074 Aachen, Germany

*Correspondence: Magnus Rueping, magnus.rueping@kaust.edu.sa

Ram Karan ram.karan@kaust.edu.sa

**Keywords:** Halophiles, extremophiles, gas vesicle, nanoparticles, biomaterials, bioengineering

**Table of Content**

| **Table S1.** Oligonucleotides used in this study  **Table S2.** Amino acid composition of GvpA  **Table S3.** Cysteine site-saturation mutagenesis within GvpA  **Figure S1:** Amino acid composition and plasmids of SpyCatcher003-mKate2 and SpyCatcher003-esterase  **Figure S2.** Arrangement of gvp genes in *Halobacterium* sp. NRC-1  **Figure S3.** Gas vesicle nanoparticles (GVNPs) engineering and expression in *Haloferax volcanii*  **Figure S4.** Gas vesicles characterization (GVNPs with GvpC vs. GvpC stripped-off)  **Figure S5.** Sequence alignment gvpA1 (gas vesicle structural protein 1) from *Halobacterium salinarum* (Uniport: P08958) and GvpA of *Bacillus megaterium* (PDB: 7R1C).  **Figure S6.** Tryptic digest and LC-MS/MS analysis of GvpA mutants and GVNP conjugates  **Figure S7.** Quenching of biotin-4-fluorescein (B4F) fluorescence by adding various amounts of pure streptavidin.  **Supplementary Discussion 1:** Cys.GvpA (N-ter)  **Supplementary Discussion 2:** GVNP morphology changes and cysteine influence  **References** | S3  S3  S4  S5  S6  S6  S7  S8  S8-S11  S12  S13  S13-S15  S-16 |
| --- | --- |

**Table S1. Oligonucleotides used in this study**

| **5′-3′sequence** | **Primer** |
| --- | --- |
| GCACCACCACCACCACCACATGCGCATAATTCAATCGATACGAGTCCCG | *Fsp*I-*Hpa*I Fwd |
| CTCGGTACCGGTTAACGGTACCGGCGGATTCTCC | *Hpa*I-*Bam*HI/*Fsp*I-*Hpa*I Rev |
| CGCCGGTACCGTTAACCGGTACCGAGGAAGAAGAGACAGAG | *Fsp*I-*Hpa*I/*Hpa*I-*Bam*HI Fwd |
| CGGCCGCTCTAGAACTAGTGGATCCGATCTGTGAGTGTACACC | *Hpa*I-*Bam*HI Rev |

**Table S2. Amino acid composition of GvpA**

Number of amino acids: 76, Molecular weight: 8005.09, Theoretical pI: 4.20

| Ala (A) | 14 | 18.4% |
| --- | --- | --- |
| Arg (R) | 3 | 3.9% |
| Asn (N) | 0 | 0.0% |
| Asp (D) | 5 | 6.6% |
| Cys (C) | 0 | 0.0% |
| Gln (Q) | 2 | 2.6% |
| Glu (E) | 9 | 11.8% |
| Gly (G) | 4 | 5.3% |
| His (H) | 1 | 1.3% |
| Ile (I) | 4 | 5.3% |
| Leu (L) | 7 | 9.2% |
| Lys (K) | 2 | 2.6% |
| Met (M) | 1 | 1.3% |
| Phe (F) | 1 | 1.3% |
| Pro (P) | 2 | 2.6% |
| Ser (S) | 4 | 5.3% |
| Thr (T) | 3 | 3.9% |
| Trp (W) | 1 | 1.3% |
| Tyr (Y) | 1 | 1.3% |

Total number of negatively charged residues (Asp + Glu): 14

Total number of positively charged residues (Arg + Lys): 5

**Table S3. Cysteine site-saturation mutagenesis within GvpA**

| **Mutant** | **Structure** |
| --- | --- |
| **Single mutants** | |
| 1.S6C | Coil |
| 2.S7C | Coil |
| 3.S47C | Loop |
| 4.A2C | Coil |
| 5.A10C | ⍺-helices |
| 6.A45C | Loop |
| 7.A46C | Loop |
| 8.A64C | ⍺-helices |
| 9.A68C | Coil |
| 10.A70C | Coil |
| 11.A72C | Coil |
| 12.A73C | Coil |
| 13.A76C | Coil |
| **Double mutants** | |
| 14. S6C.A64C | Coil, ⍺-helices |
| 15. S6C.A68C | Coil, ⍺-helices |
| 16. S6C.A70C | Coil, coil |
| 17. S6C.A72C | Coil, coil |
| 18. S6C.A73C | Coil, coil |
| 19. S6C.A76C | Coil, coil |
| 20. S7C.A64C | Coil, ⍺-helices |
| 21. S7C.A68C | Coil, ⍺-helices |
| 22. S7C.A70C | Coil, coil |
| 23. S7C.A72C | Coil, coil |
| 24. S7C.A73C | Coil, coil |
| 25. S7C.A76C | Coil, coil |
| **Triple mutants** | |
| 26. S6C.A64C.A70C | Coil, ⍺-helices, coil |
| 27. S7C.A64C.A70C | Coil, ⍺-helices, coil |
| 28. S6C.A64C.A72C | Coil, ⍺-helices, coil |
| 29. S7C.A64C.A72C | Coil, ⍺-helices, coil |
| 30. S6C.A64C.A73C | Coil, ⍺-helices, coil |
| 31. S7C.A64C.A73C | Coil, ⍺-helices, coil |
| 32. S6C.A64C.A76C | Coil, ⍺-helices, coil |
| 33. S7C.A64C.A76C | Coil, ⍺-helices, coil |
| **N- or C-terminal single mutants with a linker** | |
| Cys.GvpA (N-ter) | Cysteine with GGSGGGG linkers on the N-terminal of GvpA |
| GvpA.Cys (C-ter) | Cysteine with GGSGGGG linkers on the C-terminal of GvpA |

**SpyCatcher003-mKate2**

MSYYHHHHHHDYDIPTTENLYFQGAMVTTLSGLSGEQGPSGDMTTEEDSATHIKFSKRDEDGRELAGATMELRDSSGKTISTWISDGHVKDFYLYPGKYTFVETAAPDGYEVATPIEFTVNEDGQVTVDGEATEGDAHTGSSGSVSELIKENMHMKLYMEGTVNNHHFKCTSEGEGKPYEGTQTMRIKAVEGGPLPFAFDILATSFMYGSKTFINHTQGIPDFFKQSFPEGFTWERVTTYEDGGVLTATQDTSLQDGCLIYNVKIRGVNFPSNGPVMQKKTLGWEASTETLYPADGGLEGRADMALKLVGGGHLICNLKTTYRSKKPAKNLKMPGVYYVDRRLERIKEADKETYVEQHEVAVARYCDLPSKLGHR

**SpyCatcher003-Esterase**

MSYYHHHHHHDYDIPTTENLYFQGAMVTTLSGLSGEQGPSGDMTTEEDSATHIKFSKRDEDGRELAGATMELRDSSGKTISTWISDGHVKDFYLYPGKYTFVETAAPDGYEVATPIEFTVNEDGQVTVDGEATEGDAHTGSSGSRALLLSGCLALVLLTQQAAAQTLLVVGDSISAALGLDTSQGWVALLQKRLADEGYDYRVVNASISGDTSAGGLARLPALLAEEKPALVVIELGGNDGLRGMAPAQLQQNLASMAQKARAEGAKVLLLGIQLPPNYGPRYIEAFSRVYGAVAAQEKTALVPFFLEGVGGVQGMMQADGIHPALAAQPRLLENVWPTLKPLL


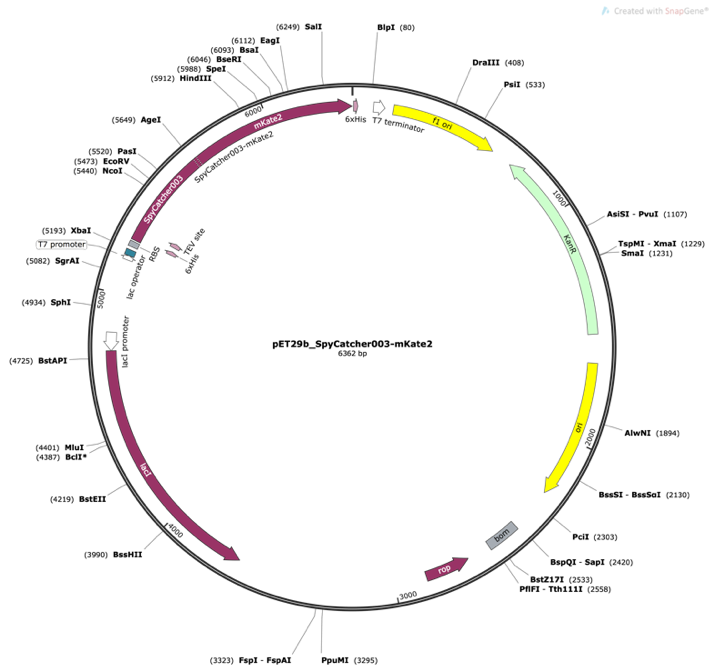

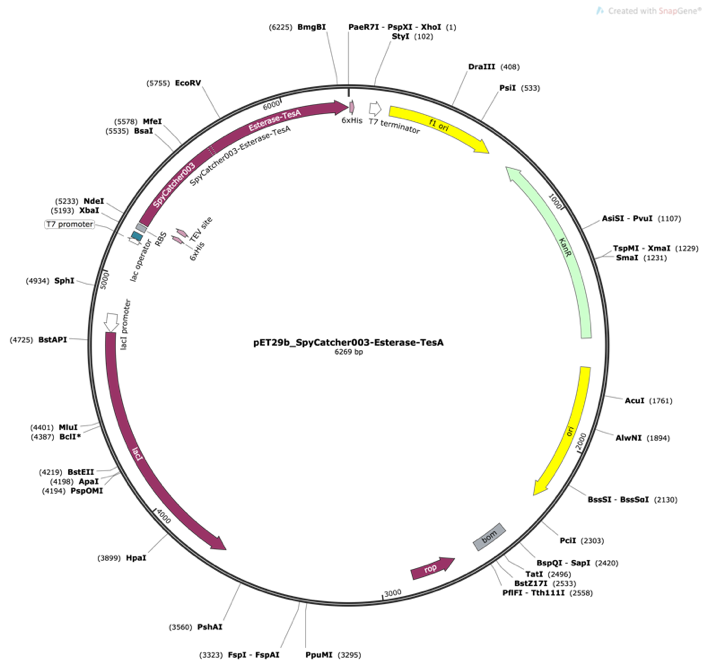


**Figure S1.** Amino acid composition and plasmids of SpyCatcher003-mKate2 (left) and SpyCatcher003-esterase (right).

**Figure S2.** **Arrangement of *gvp* genes in *Halobacterium* sp. NRC-1.** Two halobacterial gas vesicle gene clusters, gvpDEFGHIJKLM and gvpACNO have opposing transcription directions from a common origin, as indicated by the arrows.

**Figure S3. GVNPs engineering and expression in *Haloferax volcanii*. (A)** *H. volcanii* (lacking GVNPs), **(B)** Expression of GVNP from *Halobacterium* sp. NRC 1 in *H. volcanii*

**

**Figure S4. Gas vesicles characterization (GVNPs with GvpC vs. GvpC stripped-off)**

**A.** Western blot analysis indicating complete removal of GvpC protein, **B.** Straight line calibration, **C, D.** dynamic light scattering size distribution profiles of intact and partially collapsed GVNPs, **E.** Effect of temperature on diluted GVNPs. Left: GVNPs purified in PBS (GvpC stripped off), Right: 2M NaCl (containing GvpC).

pdb|7r1c|N MSIQKSTNSSSLAEVIDRILDKGIVIDAFARVSVVGIEILTIEARVVIASVDTWLRYAEA 60

GvpA NRC-1 ---MAQPDSSGLAEVLDRVLDKGVVVDVWARVSLVGIEILTVEARVVAASVDTFLHYAEE 57

. :**.****:**:****:*:*.:****:*******:***** *****:*:***

pdb|7r1c|N VGLLRDDVEENGLPERSNSSEGQPRFSI 88

GvpA NRC-1 IAKIEQAELTAGAEAAPEA--------- 76

:. :.: * ::

**Figure S5.** Sequence alignment gvpA1 (gas vesicle structural protein 1) from *Halobacterium salinarum* (strain ATCC 700922/JCM 11081/NRC-1, Uniport: P08958) and GvpA of *Bacillus megaterium* (PDB: 7R1C).

**1.GvpA.S6C**

MAQPDCSGLAEVLDRVLDKGVVVDVWARVSLVGIEILTVEARVVAASVDTFLHYAEEIAKIEQAELTAGAEAAPEA

**2.GvpA.S7C**

MAQPDSCGLAEVLDRVLDKGVVVDVWARVSLVGIEILTVEARVVAASVDTFLHYAEEIAKIEQAELTAGAEAAPEA

**3.GvpA.S47C**

MAQPDSSGLAEVLDRVLDKGVVVDVWARVSLVGIEILTVEARVVAACVDTFLHYAEEIAKIEQAELTAGAEAAPEA

**4.GvpA.A2C**

MCQPDSSGLAEVLDRVLDKGVVVDVWARVSLVGIEILTVEARVVAASVDTFLHYAEEIAKIEQAELTAGAEAAPEA

**5.GvpA.A10C**

MAQPDSSGLCEVLDRVLDKGVVVDVWARVSLVGIEILTVEARVVAASVDTFLHYAEEIAKIEQAELTAGAEAAPEA

**6.GvpA.A45C**

MAQPDSSGLAEVLDRVLDKGVVVDVWARVSLVGIEILTVEARVVCASVDTFLHYAEEIAKIEQAELTAGAEAAPEA

**7.GvpA.A46C**

MAQPDSSGLAEVLDRVLDKGVVVDVWARVSLVGIEILTVEARVVACSVDTFLHYAEEIAKIEQAELTAGAEAAPEA

**8.GvpA.A63C**

MAQPDSSGLAEVLDRVLDKGVVVDVWARVSLVGIEILTVEARVVAASVDTFLHYAEEIAKIEQCELTAGAEAAPEA

**9.GvpA.A68C**

MAQPDSSGLAEVLDRVLDKGVVVDVWARVSLVGIEILTVEARVVAASVDTFLHYAEEIAKIEQAELTCGAEAAPEA

**10.GvpA.A70C**

MAQPDSSGLAEVLDRVLDKGVVVDVWARVSLVGIEILTVEARVVAASVDTFLHYAEEIAKIEQAELTAGCEAAPEA

**11.GvpA.A72C**

MAQPDSSGLAEVLDRVLDKGVVVDVWARVSLVGIEILTVEARVVAASVDTFLHYAEEIAKIEQAELTAGAECAPEA

**12.GvpA.A73C**

MAQPDSSGLAEVLDRVLDKGVVVDVWARVSLVGIEILTVEARVVAASVDTFLHYAEEIAKIEQAELTAGAEACPEA

**13.GvpA.A76C**

MAQPDSSGLAEVLDRVLDKGVVVDVWARVSLVGIEILTVEARVVAASVDTFLHYAEEIAKIEQAELTAGAEAAPEC

**14.GvpA.S6C.A63C**
MAQPDCSGLAEVLDRVLDKGVVVDVWARVSLVGIEILTVEARVVAASVDTFLHYAEEIAKIEQCELTAGAEAAPEA

**15.GvpA.S6C.A68C**
MAQPDCSGLAEVLDRVLDKGVVVDVWARVSLVGIEILTVEARVVAASVDTFLHYAEEIAKIEQAELTCGA EAAPEA

**16.GvpA.S6C.A70C**
MAQPDCSGLAEVLDRVLDKGVVVDVWARVSLVGIEILTVEARVVAASVDTFLHYAEEIAKIEQAELTAGCEAAPEA

**17.GvpA.S6C.A72C**
MAQPDCSGLAEVLDRVLDKGVVVDVWARVSLVGIEILTVEARVVAASVDTFLHYAEEIAKIEQAELTAGAECAPEA

**18.GvpA.S6C.A73C**
MAQPDCSGLAEVLDRVLDKGVVVDVWARVSLVGIEILTVEARVVAASVDTFLHYAEEIAKIEQAELTAGAEACPEA

**19.GvpA.S6C.A76C**
MAQPDCSGLAEVLDRVLDKGVVVDVWARVSLVGIEILTVEARVVAASVDTFLHYAEEIAKIEQAELTAGAEAAPEC

**20.GvpA.S7C.A63C**
MAQPDSCGLAEVLDRVLDKGVVVDVWARVSLVGIEILTVEARVVAASVDTFLHYAEEIAKIEQCELTAGAEAAPEA

**21.GvpA.S7C.A68C**
MAQPDSCGLAEVLDRVLDKGVVVDVWARVSLVGIEILTVEARVVAASVDTFLHYAEEIAKIEQAELTCGAEAAPEA

**22.GvpA.S7C.A70C**
MAQPDSCGLAEVLDRVLDKGVVVDVWARVSLVGIEILTVEARVVAASVDTFLHYAEEIAKIEQAELTAGCEAAPEA

**23.GvpA.S7C.A72C**
MAQPDSCGLAEVLDRVLDKGVVVDVWARVSLVGIEILTVEARVVAASVDTFLHYAEEIAKIEQAELTAGAECAPEA

**24.GvpA.S7C.A73C**
MAQPDSCGLAEVLDRVLDKGVVVDVWARVSLVGIEILTVEARVVAASVDTFLHYAEEIAKIEQAELTAGAEACPEA

**25.GvpA.S7C.A76C**
MAQPDSCGLAEVLDRVLDKGVVVDVWARVSLVGIEILTVEARVVAASVDTFLHYAEEIAKIEQAELTAGAEAAPEC

**26.GvpA.S6C.A63C.A70C**
MAQPDCSGLAEVLDRVLDKGVVVDVWARVSLVGIEILTVEARVVAASVDTFLHYAEEIAKIEQCELTAGCEAAPEA

**27.GvpA.S7C.A63C.A70C**
MAQPDSCGLAEVLDRVLDKGVVVDVWARVSLVGIEILTVEARVVAASVDTFLHYAEEIAKIEQCELTAGCEAAPEA

**28.GvpA.S6C.A63C.A72C**
MAQPDCSGLAEVLDRVLDKGVVVDVWARVSLVGIEILTVEARVVAASVDTFLHYAEEIAKIEQCELTAGAECAPEA

**29.GvpA.S7C.A63C.A72C**
MAQPDSCGLAEVLDRVLDKGVVVDVWARVSLVGIEILTVEARVVAASVDTFLHYAEEIAKIEQCELTAGAECAPEA

**30.GvpA.S6C.A63C.A73C**
MAQPDCSGLAEVLDRVLDKGVVVDVWARVSLVGIEILTVEARVVAASVDTFLHYAEEIAKIEQCELTAGAEACPEA

**31.GvpA.S7C.A63C.A73C**
MAQPDSCGLAEVLDRVLDKGVVVDVWARVSLVGIEILTVEARVVAASVDTFLHYAEEIAKIEQCELTAGAEACPEA

**32.GvpA.S6C.A63C.A76C**
MAQPDCSGLAEVLDRVLDKGVVVDVWARVSLVGIEILTVEARVVAASVDTFLHYAEEIAKIEQCELTAGAEAAPEC

**33.GvpA.S7C.A63C.A76C**
MAQPDSCGLAEVLDRVLDKGVVVDVWARVSLVGIEILTVEARVVAASVDTFLHYAEEIAKIEQCELTAGAEAAPEC

**GvpA_C.malSpyTag.SpyCatcher_mKate**

MAQPDSSGLAEVLDRVLDKGVVVDVWARVSLVGIEILTVEARVVAASVDTFLHYAEEIAKIEQAELTAGAEAAPEAGGSGGGGCGGSGGSGRGVPHIVMVDAYKRYKMSYYHHHHHHDYDIPTTENLYFQGAMVTTLSGLSGEQGPSGDMTTEEDSATHIKFSKRDEDGRELAGATMELRDSSGKTISTWISDGHVKDFYLYPGKYTFVETAAPDGYEVATPIEFTVNEDGQVTVDGEATEGDAHTGSSGSVSELIKENMHMKLYMEGTVNNHHFKCTSEGEGKPYEGTQTMRIKAVEGGPLPFAFDILATSFMYGSKTFINHTQGIPDFFKQSFPEGFTWERVTTYEDGGVLTATQDTSLQDGCLIYNVKIRGVNFPSNGPVMQKKTLGWEASTETLYPADGGLEGRADMALKLVGGGHLICNLKTTYRSKKPAKNLKMPGVYYVDRRLERIKEADKETYVEQHEVAVARYCDLPSKLGHR

**GvpA_TM28.malSpyTag.SpyCatcher_mKate**

MAQPDCSGLAEVLDRVLDKGVVVDVWARVSLVGIEILTVEARVVAASVDTFLHYAEEIAKIEQCELTAGAECAPEAGGSGGSGRGVPHIVMVDAYKRYKMSYYHHHHHHDYDIPTTENLYFQGAMVTTLSGLSGEQGPSGDMTTEEDSATHIKFSKRDEDGRELAGATMELRDSSGKTISTWISDGHVKDFYLYPGKYTFVETAAPDGYEVATPIEFTVNEDGQVTVDGEATEGDAHTGSSGSVSELIKENMHMKLYMEGTVNNHHFKCTSEGEGKPYEGTQTMRIKAVEGGPLPFAFDILATSFMYGSKTFINHTQGIPDFFKQSFPEGFTWERVTTYEDGGVLTATQDTSLQDGCLIYNVKIRGVNFPSNGPVMQKKTLGWEASTETLYPADGGLEGRADMALKLVGGGHLICNLKTTYRSKKPAKNLKMPGVYYVDRRLERIKEADKETYVEQHEVAVARYCDLPSKLGHR

**GvpA_C.malSpyTag.SpyCatcher_SuperNova**

MAQPDSSGLAEVLDRVLDKGVVVDVWARVSLVGIEILTVEARVVAASVDTFLHYAEEIAKIEQAELTAGAEAAPEAGGSGGGGCGGSGGSGRGVPHIVMVDAYKRYKMSYYHHHHHHDYDIPTTENLYFQGAMVTTLSGLSGEQGPSGDMTTEEDSATHIKFSKRDEDGRELAGATMELRDSSGKTISTWISDGHVKDFYLYPGKYTFVETAAPDGYEVATPIEFTVNEDGQVTVDGEATEGDAHTGSSGSEVGPALFQSDMTFKIFIDGEVNGQKFTIVADGSSKFPHGDFNVHAVCETGKLPMSWKPICHLIQYGEPFFARYPDGISHFAQECFPEGLSIDRTVRFENDGTMTSHHTYELDDTCVVSRITVNCDGFQPDGPIMRDQLVDILPSETHMFPHGPNAVRQTATIGFTTADGGKMMGHFDSKMTFNGSRAIEIPGPHFVTIITKQTRDTSDKRDHVCQREVAYAHSVPRITSAIGSDED

**GvpA_TM28.malSpyTag.SpyCatcher_SuperNova**

MAQPDCSGLAEVLDRVLDKGVVVDVWARVSLVGIEILTVEARVVAASVDTFLHYAEEIAKIEQCELTAGAECAPEAGGSGGSGRGVPHIVMVDAYKRYKMSYYHHHHHHDYDIPTTENLYFQGAMVTTLSGLSGEQGPSGDMTTEEDSATHIKFSKRDEDGRELAGATMELRDSSGKTISTWISDGHVKDFYLYPGKYTFVETAAPDGYEVATPIEFTVNEDGQVTVDGEATEGDAHTGSSGSEVGPALFQSDMTFKIFIDGEVNGQKFTIVADGSSKFPHGDFNVHAVCETGKLPMSWKPICHLIQYGEPFFARYPDGISHFAQECFPEGLSIDRTVRFENDGTMTSHHTYELDDTCVVSRITVNCDGFQPDGPIMRDQLVDILPSETHMFPHGPNAVRQTATIGFTTADGGKMMGHFDSKMTFNGSRAIEIPGPHFVTIITKQTRDTSDKRDHVCQREVAYAHSVPRITSAIGSDED

**GvpA_C.malSpyTag.SpyCatcher_Esterase**

MAQPDSSGLAEVLDRVLDKGVVVDVWARVSLVGIEILTVEARVVAASVDTFLHYAEEIAKIEQAELTAGAEAAPEAGGSGGGGCGGSGGSGRGVPHIVMVDAYKRYKMSYYHHHHHHDYDIPTTENLYFQGAMVTTLSGLSGEQGPSGDMTTEEDSATHIKFSKRDEDGRELAGATMELRDSSGKTISTWISDGHVKDFYLYPGKYTFVETAAPDGYEVATPIEFTVNEDGQVTVDGEATEGDAHTGSSGSRALLLSGCLALVLLTQQAAAQTLLVVGDSISAALGLDTSQGWVALLQKRLADEGYDYRVVNASISGDTSAGGLARLPALLAEEKPALVVIELGGNDGLRGMAPAQLQQNLASMAQKARAEGAKVLLLGIQLPPNYGPRYIEAFSRVYGAVAAQEKTALVPFFLEGVGGVQGMMQADGIHPALAAQPRLLENVWPTLKPLL

**GvpA_C.malStreptavidin**

MAQPDSSGLAEVLDRVLDKGVVVDVWARVSLVGIEILTVEARVVAASVDTFLHYAEEIAKIEQAELTAGAEAAPEAGGSGGGGCMRKIVVAAIAVSLTTVSITASASADPSKDSKAQVSAAEAGITGTWYNQLGSTFIVTAGADGALTGTYESAVGNAESRYVLTGRYDSAPATDGSGTALGWTVAWKNNYRNAHSATTWSGQYVGGAEARINTQWLLTSGTTEANAWKSTLVGHDTFTKVKPSAASIDAAKKAGVNNGNPLDAVQQ

**GvpA_C.malStrep_ Biotin-HRP**

MAQPDSSGLAEVLDRVLDKGVVVDVWARVSLVGIEILTVEARVVAASVDTFLHYAEEIAKIEQAELTAGAEAAPEAGGSGGGGCMRKIVVAAIAVSLTTVSITASASADPSKDSKAQVSAAEAGITGTWYNQLGSTFIVTAGADGALTGTYESAVGNAESRYVLTGRYDSAPATDGSGTALGWTVAWKNNYRNAHSATTWSGQYVGGAEARINTQWLLTSGTTEANAWKSTLVGHDTFTKVKPSAASIDAAKKAGVNNGNPLDAVQQMHFSSSSTLFTCITLIPLVCLILHASLSDAQLTPTFYDNSCPNVSNIVRDTIVNELRSDPRIAASILRLHFHDCFVNGCDASILLDNTTSFRTEKDAFGNANSARGFPVIDRMKAAVESACPRTVSCADLLTIAAQQSVTLAGGPSWRVPLGRRDSLQAFLDLANANLPAPFFTLPQLKDSFRNVGLNRSSDLVALSGGHTFGKNQCRFIMDRLYNFSNTGLPDPTLNTTYLQTLRGLCPLNGNLSALVDFDLRTPTIFDNKYYVNLEEQKGLIQSDQELFSSPNATDTIPLVRSFANSTQTFFNAFVEAMDRMGNITPLTGTQGQIRLNCRVVNSNSLLHDMVEVVDFVSSM

**Figure S6. Tryptic digest and LC-MS/MS analysis of GvpA mutants and GVNP conjugates.** Matched peptides are underlined*.*

**Figure S7. Standard Curve of biotin-4-fluorescein Assay.** Quenching of biotin-4-fluorescein (B4F) fluorescence by adding various amounts of pure streptavidin. Fluorescence (485/525 nm) was read after a 10-min incubation.

**Supplementary Discussion 1: C.GvpA (N-ter)**

The C.GvpA (N-ter) variant was not expressed and could not be detected by tryptic digest and LC-MS/MS analysis. We assume that the N-terminal extension changes the folding mechanism of the GvpA variant and destabilizes the protein and thus causing the denaturation of the C_GvpA (N-ter) variant. Several studies showed that the length and sequence of the N-terminal extension could adversely affect the protein's stability and kinetic-folding pathway.^1-3^ Since GvpA is the main building block of GVNPs and forms the essential core of the structure, it is necessarily required to form GVNP.^4^ Therefore, the non-production of GvpA or any GvpA variants abolishes the GVNP formation.

**Supplementary Discussion 2: GVNP morphology changes and cysteine influence**

To date, the biogenesis and assembly of wild-type GVNPs are not fully understood and are still a 'black box.' Furthermore, even in wild-type GVNPs, different morphologies are reported.^5, 6^ Simon *et al*. reported that *Halobacterium salinarium* wild-type strains produced a majority of spindle-shaped GVNPs (94 %). In comparison, 69% of the GVNPs were cylindrical in gas vacuole defective isolates.^5^

The fact that two different morphologies result from a single polypeptide type would suggest that factors other than the nature of the polypeptide subunit, GvpA, must be involved. Thus, the current hypothesis is that polypeptide processing may be involved in the formation and assembly of gas vesicles. We observed in our study that specific GvpA variants displayed an elongation effect in the spindle-shaped GVNPs. These elongated spindle-shaped GVNPs can also be observed in wild-type GVNPs. Moreover, the accurate structure of the GVNP subunit GvpA and the assembly interface was still unknown until 2022. Recently, two groups investigated the structure of GVNPs by cryo-EM and cryo-ET and successfully reconstructed GVNP assemblies from *B. megaterium* and *Anabaena flos-aquae*.^6, 7^ Huber *et al.* reported a pseudo-atomic model of an entire GVNP particle (Figure S8) and established the molecular basis to understand further processes in the biogenesis of GVNPs such as nucleation, growth, width regulation, and function of other GVNP gene products in GVNP assembly.^6^ However, these research questions require further investigation.


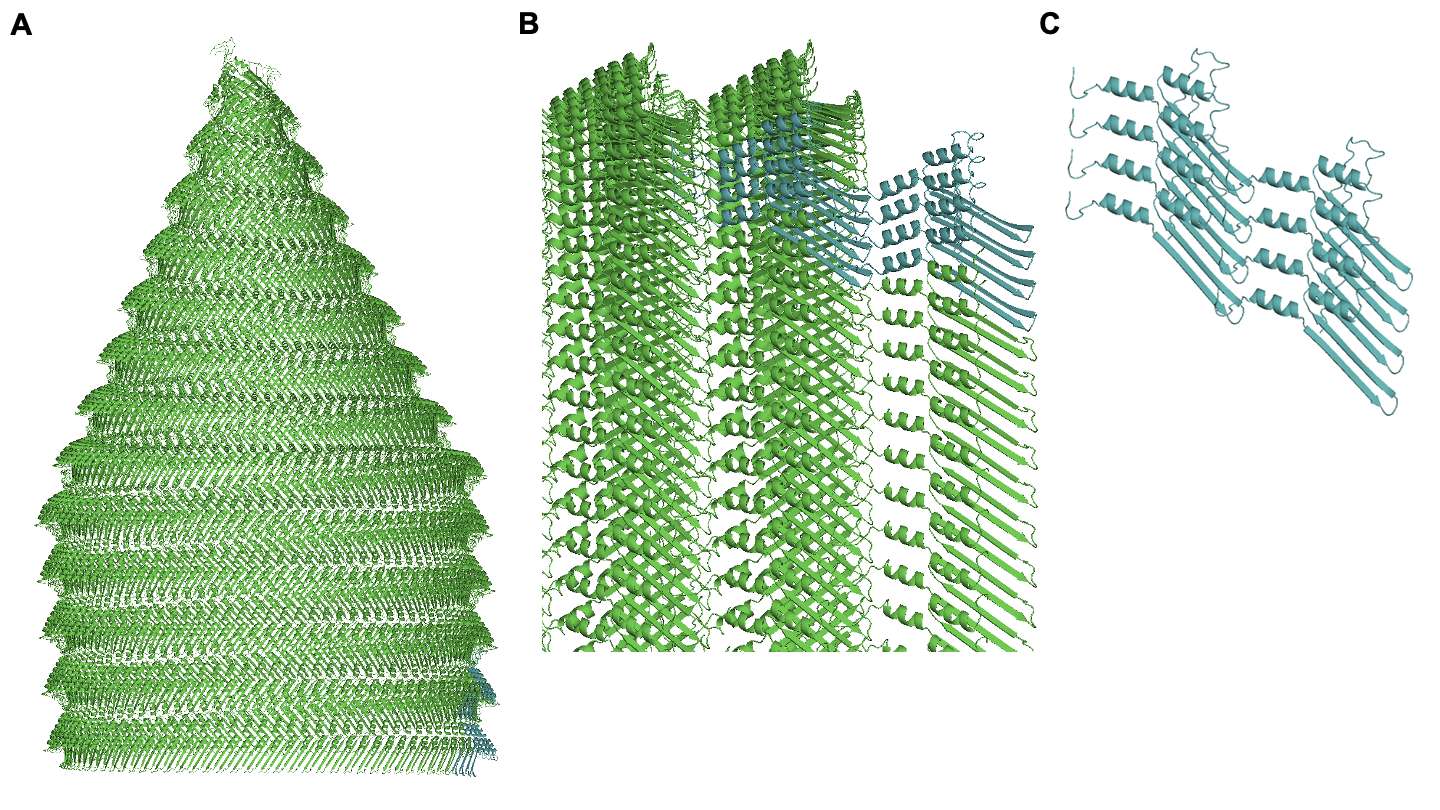


**Figure S8.** **Pseudo-atomic model of an entire GVNP particle**.^6^ (A) Pseudo-atomic model of GVNP, (B) Close-up on rib structure of GvpA subunits, and (C) GvpA assembly interface. GvpA subunits are displayed in cartoon representation. The assembly interface of four GvpA units is highlighted in cyan.

We used these now available datasets^6, 7^ to understand why some cysteine variants did not form GVNPs. However, we would like to point out that it is speculative. Since tryptic digest and LC-MS/MS analysis confirmed the presence of the monomer subunits (A2C, A10C, S7C.A64C, S7C.A76C, S7CA.64C.A70C, S7CA.64C.A72C, S7CA.64C.A73C, and S7CA.64C.A76C), we hypothesize that the cysteine substitutions are interfering with the assembly interface, and thus not allow the formation of GVNPs. The 'Cys.GvpA (N-ter)' variant could not be detected by tryptic digest and LC-MS/MS analysis, and we assume the N-terminal insertion affects the protein folding.

We did not observe GVNP formation for variants A2C, A10C, S7C.A64C, S7C.A76C, S7CA.64C.A70C, S7CA.64C.A72C, S7CA.64C.A73C, and S7CA.64C.A76C (Figure S9). In the GvpA variants A2C and A10C (Figure S9A, B), the cysteines of each subunit seem to be able to potentially form disulfide bonds in their flexible regions (mainly between 3.0 Å and 7.5 Å)^8^ and thus forming a ridge breaking/ steric hindrance motif. Similarly, in the GvpA variants S7C.A64C and S7C.A76C (Figure S9C, D), the corresponding cysteines in the subunits can come close and potentially form disulfide bonds. Since the GvpA variants S7C.A64C and S7C.A76C cannot form GVNPs, it makes sense that the logically build-up triple cysteine mutants S7CA.64C.A70C, S7CA.64C.A72C, S7CA.64C.A73C, and S7CA.64C.A76C are not assembling as well.


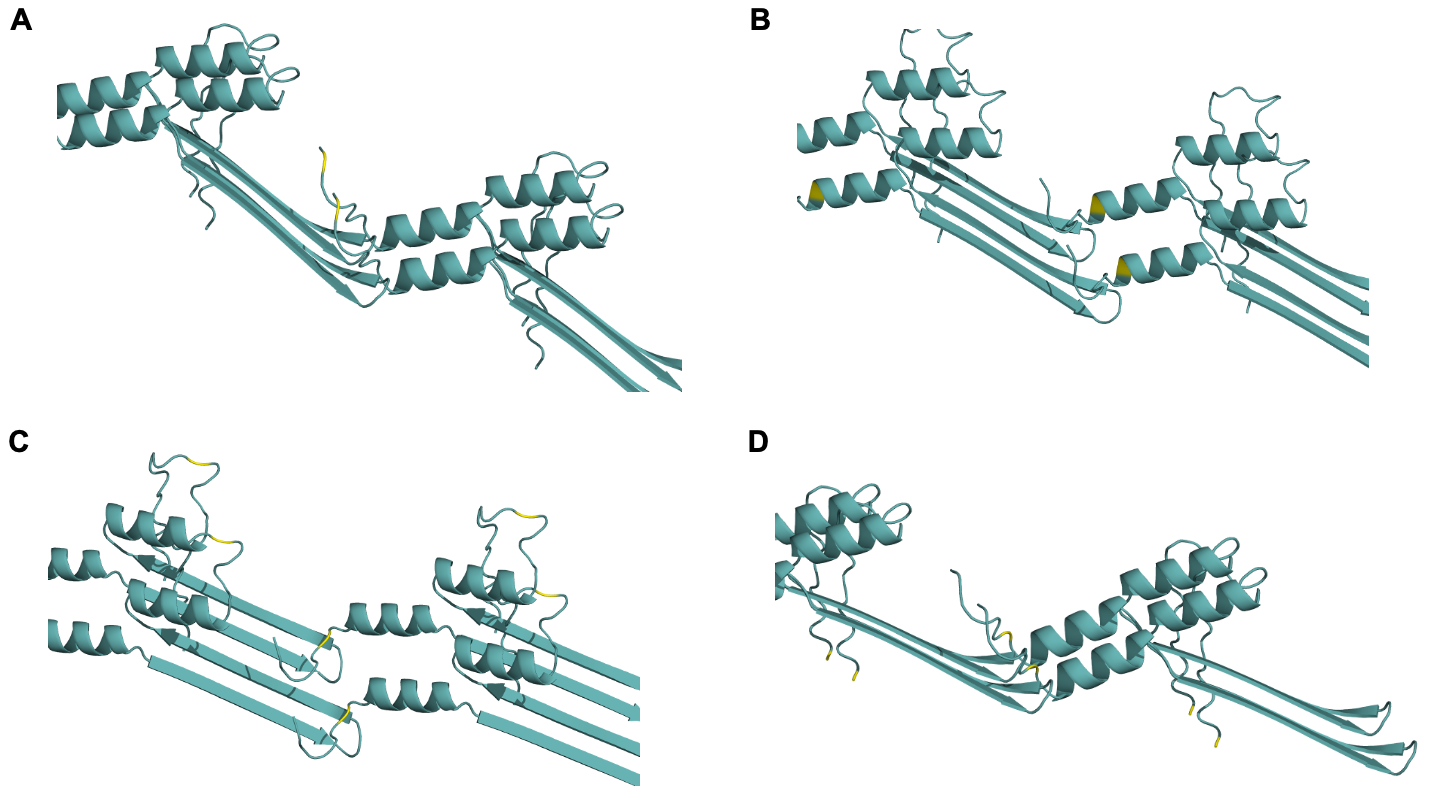


**Figure S9 GVNP subunit with GvpA models based on the pseudo-atomic model of an entire GVNP particle by Huber *et al***.^6^ (A) Assembly interface of GvpA A2C variant, (B) assembly interface of GvpA A10C variant, (C) assembly interface of GvpA S7C.A64C variant, (D) assembly interface of GvpA S7C.A76C variant. The cysteine insertion positions are highlighted in yellow.

**References**

1. Korepanova, A.; Douglas, C.; Leyngold, I.; Logan, T. M., N-terminal extension changes the folding mechanism of the FK506-binding protein. *Protein Science* **2001,** *10* (9), 1905-1910.

2. Liu, K.; Chen, X.; Kaiser, C. M., Energetic dependencies dictate folding mechanism in a complex protein. *Proceedings of the National Academy of Sciences* **2019,** *116* (51), 25641-25648.

3. Chang, L.; Perez, A., Deciphering the Folding Mechanism of Proteins G and L and Their Mutants. *Journal of the American Chemical Society* **2022,** *144* (32), 14668-14677.

4. Pfeifer, F. Recent Advances in the Study of Gas Vesicle Proteins and Application of Gas Vesicles in Biomedical Research *Life* [Online], 2022.

5. Simon, R. D., Morphology and Protein Composition of Gas Vesicles from Wild Type and Gas Vacuole Defective Strains of Halobacterium salinarium Strain 5. *Microbiology* **1981,** *125* (1), 103-111.

6. Huber, S. T.; Terwiel, D.; Evers, W. H.; Maresca, D.; Jakobi, A. J., Cryo-EM structure of gas vesicles for buoyancy-controlled motility. *Cell* **2023,** *186* (5), 975-986.e13.

7. Dutka, P.; Metskas, L. A.; Hurt, R. C.; Salahshoor, H.; Wang, T.-Y.; Malounda, D.; Lu, G.; Chou, T.-F.; Shapiro, M. G.; Jensen, G. J., Structure of &lt;em&gt;Anabaena flos-aquae&lt;/em&gt; gas vesicles revealed by cryo-ET. *bioRxiv* **2022**, 2022.06.21.496981.

8. Gao, X.; Dong, X.; Li, X.; Liu, Z.; Liu, H., Prediction of disulfide bond engineering sites using a machine learning method. *Scientific Reports* **2020,** *10* (1), 10330.
